# Action in auctions: neural and computational mechanisms of bidding behaviour

**DOI:** 10.1111/ejn.14492

**Published:** 2019-07-29

**Authors:** Mario Martinez-Saito, Rodion Konovalov, Michael A. Piradov, Anna Shestakova, Boris Gutkin, Vasily Klucharev

**Affiliations:** ^1^ Centre for Cognition and Decision Making Institute of Cognitive Neuroscience National Research University Higher School of Economics Moscow Russian Federation; ^2^ Research Center of Neurology Moscow Russian Federation; ^3^ Group for Neural Theory LNC INSERM U960 PSL* Research University Ecole Normale Superieure Paris France

**Keywords:** adaptive learning, internal number line, social competition, striatum, value‐based decision‐making

## Abstract

Competition for resources is a fundamental characteristic of evolution. Auctions have been widely used to model competition of individuals for resources, and bidding behaviour plays a major role in social competition. Yet, how humans learn to bid efficiently remains an open question. We used model‐based neuroimaging to investigate the neural mechanisms of bidding behaviour under different types of competition. Twenty‐seven subjects (nine male) played a prototypical bidding game: a double action, with three “market” types, which differed in the number of competitors. We compared different computational learning models of bidding: directional learning models (DL), where the model bid is “nudged” depending on whether it was accepted or rejected, along with standard reinforcement learning models (RL). We found that DL fit the behaviour best and resulted in higher payoffs. We found the binary learning signal associated with DL to be represented by neural activity in the striatum distinctly posterior to a weaker reward prediction error signal. We posited that DL is an efficient heuristic for valuation when the action (bid) space is continuous. Indeed, we found that the posterior parietal cortex represents the continuous action space of the task, and the frontopolar prefrontal cortex distinguishes among conditions of social competition. Based on our findings, we proposed a conceptual model that accounts for a sequence of processes that are required to perform successful and flexible bidding under different types of competition.

## Graphical abstract

Maximizing buyer payoff in a simple auction of one seller versus one buyer: a RL strategy picks bids based on an account of the value of each bid, whereas a DL strategy simply nudges up and down the bid contingent on previous feedback.

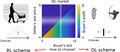

**Abbreviations**
BC: buyer competition market
DL: directional learning
DS: directional signature
fMRI: functional magnetic resonance Imaging
MDI: market discrimination index
NC: no competition market
PBV: preferred bid value
PFC: prefrontal cortex
PPC: posterior parietal cortex
RL: reinforcement learning
RPE: reward prediction error
RW: Rescorla–Wagner
SC: seller competition market

## 1 INTRODUCTION

We often deal with situations where buyers and sellers meet to exchange goods at prices determined by fluctuations in supply and demand. Perceived market competition influences human bidding (van den Bos et al., 2008; Fischbacher, Fong, & Fehr, 2009) and even the value of commodities traded by non‐human animals. For instance, baboons (Henzi & Barrett, 2002) and vervet monkeys (Fruteau, Voelkl, van Damme, & Noë, 2009) demonstrate the effect of market competition on the price of natural currencies such as food or grooming. Indeed, biological auctions are used to model competition between species and individuals (Reiter, Kanodia, Gupta, Nowak, & Chatterjee, 2015). Despite its key importance in social behaviour and financial modelling, the neural mechanisms of decision‐making under market competition are still unclear. In particular, how do we learn bidding strategies across different market scenarios? Here, we investigate the neural mechanisms underlying bidding under different conditions of competition.

The study of bidding behaviour lies at the intersection of behavioural economics, game theory and cognitive neuroscience. Much previous research has focused on simple sequential game theoretic paradigms, such as the ultimatum game (UG; Güth, Schmittberger, & Schwarze, 1982; Sanfey, Rilling, Aronson, Nystrom, & Cohen, 2003). Behavioural studies have shown that competition in UGs among proposers leads to higher bid offers (Roth, Prasnikar, Okuno‐Fujiwara, & Zamir, 1991), and in general, it pushes players towards Nash equilibria with tell‐tale lower rejection rates (Fischbacher et al., 2009). A combination of fairness concerns and decision errors has been put forward to explain the effect of competition on offer distributions in UGs (Fischbacher et al., 2009), but it is not clear how offers are picked in more general settings. In simultaneous bidding paradigms, quantal response equilibrium (McKelvey & Palfrey, 1995), a normative solution concept from game theory, has been shown to capture behaviour well. However, this model offers little insight into biological learning mechanisms and requires costly computations based on beliefs about other players. In repeated games, players typically demonstrate an extended adaptation to the environment conditions (Fudenberg & Levine, 1998; Grosskopf, 2003; Roth et al., 1991), and very simple models have been shown to perform robustly as long as enough information about other players is provided (Fudenberg & Levine, 2009). Moreover, behavioural economics experiments show that adaptive learning algorithms explain bargaining behaviour well (Camerer & Ho, 1999; Erev & Roth, 1998; Mookherjee & Sopher, 1994). Thus, a parsimonious learning model should be suitable for explaining offer distributions under changing supply and demand conditions.

Previous neuroimaging studies investigated bargaining games, but focused on strategic deception and uncertainty about trustworthiness (Bhatt, Lohrenz, Camerer, & Montague, 2010, 2012) or examined the influence of loss contemplation under social contexts in overbidding (Delgado, Schotter, Ozbay, & Phelps, 2008). In this study, we investigated the neural mechanism of bidding behaviour under different conditions of competition. Subjects played the role of buyers in a double auction in three different market types, which differed in levels of supply and demand. To investigate buyer's decisions, we set the transaction price to equal the buyer's bid, which in case of acceptance becomes the final price, while rejection was set to be the worst outcome. This paradigm is similar to online auctions such as eBay auction, where multiple buyers bid for a good, and in financial transactions with buy limit orders (assuming that buyers are strongly incentivized to acquire the security/good). In these scenarios, repeated bidding serves to “probe” the market and estimates its current clearing price in a trial‐and‐error fashion, and whereby, the buyer learns to bid more efficiently given the estimated clearing price and her needs.

Although traditionally theoretical accounts of adaptive learning in decision‐making tend to focus on model‐free reinforcement learning (RL), algorithms that are beyond this minimal account may be more suitable for bidding. One such framework that is particularly suitable for bidding, directional learning (DL), suggests a simple adaptive strategy that takes into account that the available bids are ordered consistently (Selten & Buchta, 1994) and requires a representation of a one‐dimensional continuum. According to DL, profitable bids exhibit a simple Markovian dependence on the immediately previous outcome: it is adjusted up (down) if it was too low (high) in the previous period.

To our knowledge, DL models have not been used in neuroimaging studies to probe the neural correlates of economic decision‐making. However, numerous functional magnetic resonance imaging (fMRI) studies have shown that RL operational variables, such as expected value and reward prediction error (RPE), can be used to trace neural correlates of adaptive learning (e.g., Montague, King‐Casas, & Cohen, 2006; Ruff & Fehr, 2014). For example, neural correlates of RPE have repeatedly been located in the ventromedial prefrontal cortex (vmPFC) and the ventral striatum (Bartra, McGuire, & Kable, 2013; O'Doherty et al., 2004). But, such studies often use relatively simple decision‐making tasks, structured specifically to be solvable by RL in a reasonable time, often with discrete response policies, while economic tasks involving continuous decision variables and policies that need to be structured over such real‐value scales have been explored to a lesser extent. Here, we focus specifically on the neural underpinnings of DL and RL strategies that drive repeated bidding behaviour under different types of buyer/seller competition.

## 2 MATERIALS AND METHODS

### 2.1 Subjects

Twenty‐seven subjects (nine males, two left‐handed, after discarding two of the initial 29 subjects due to excessive head motion) took part in the experiment. All subjects were queried to exclude histories of neurological pathologies. After a briefing, all subjects gave informed written consent and paid upon completion of the task. The protocol was performed in accordance with the Declaration of Helsinki with approval of the University Review Board of Higher School of Economics.

### 2.2 The double auction paradigm

**Figure 1:**
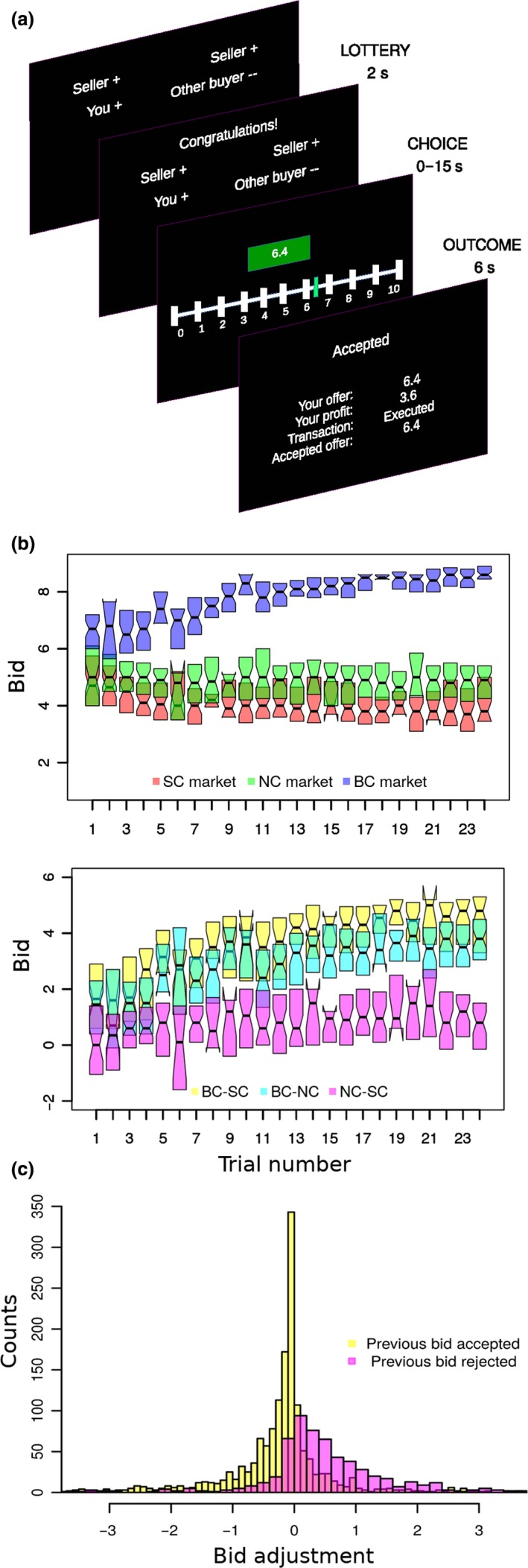
Task design and behavioural results. (a) Each trial consisted of four stages: market type announcement, lottery, bid selection and game outcome feedback. During the market announcement stage (MARKET), the subject was informed of the market type of the current trial. The next stage (LOTTERY) indicated whether the subject would go forth to the next stage or be redirected to the beginning of the next trial. In the former case, a Likert scale was displayed, and the subject had to choose her bid by sliding a vertical bar (CHOICE). Finally, the game outcome stage (OUTCOME) signalled whether the bid was accepted (ACCEPTED) or rejected (REJECTED). (b) Upper: behavioural learning dynamics of bids across all subjects. Lower: pairwise differences of bid sizes among market types. Box “hinges” represent first and third quartiles. (c) Bid adjustments were contingent on the previous trial's outcome of the same market type. [Colour figure can be viewed at http://wileyonlinelibrary.com]

To probe neural mechanisms of bid learning, we used a modified version of the double auction, a standard paradigm in multiplayer game theory where players try to maximize their respective benefit by means of a single‐shot transaction (Fudenberg & Tirole, 1991). Subjects played the role of buyers in a double auction with first‐price sealed bids and with opponents assigned by repeated random matching, in three different market types (Figure 1a).

**Figure 2:**
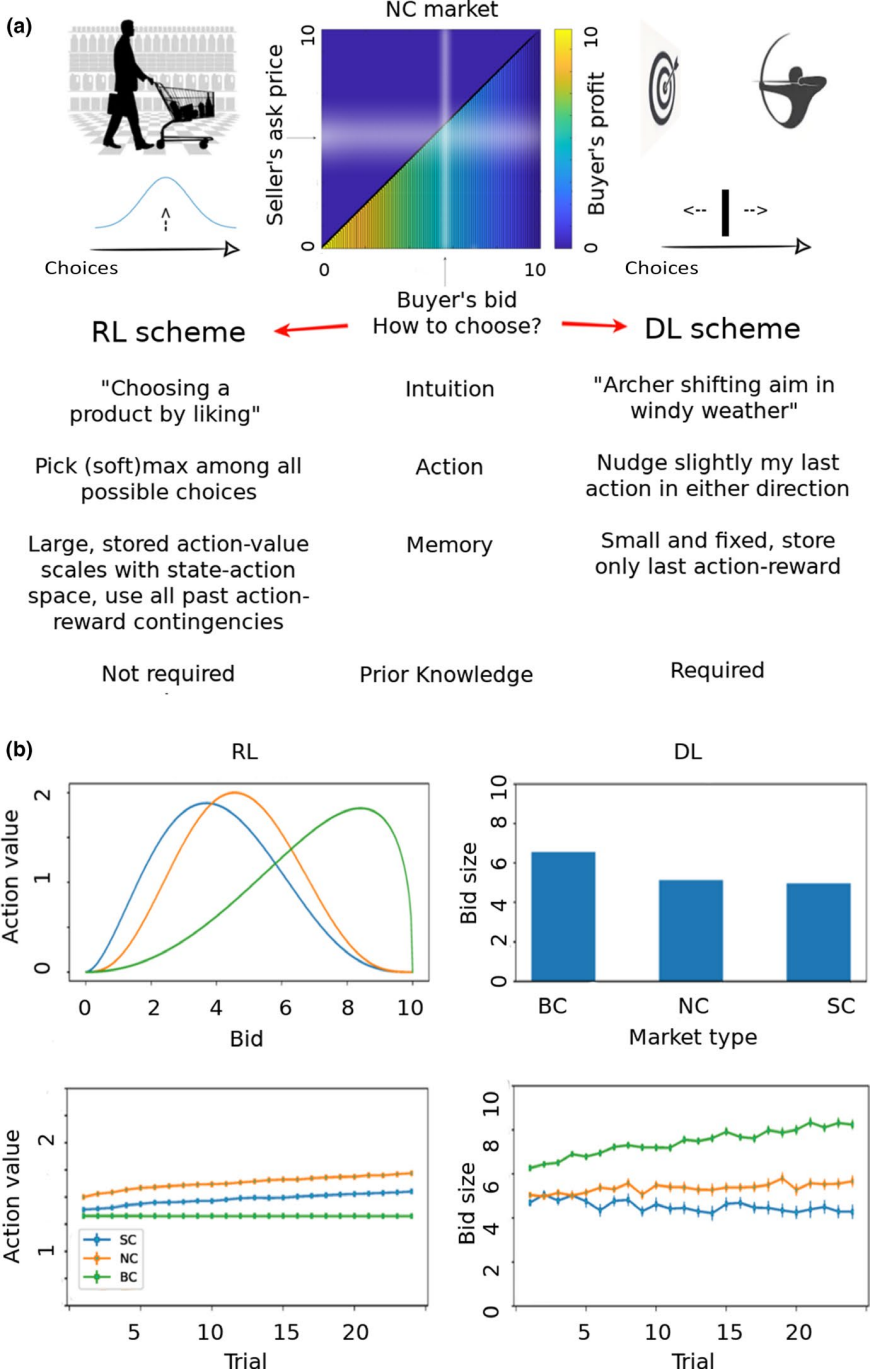
RL‐ and DL‐type algorithms comparison. (a) Normal form (top centre) of a one seller versus one buyer (NC market) game: matrix cell colours represent the buyer's payoff. The buyer holds an estimate of the (possibly varying) seller's ask price (horizontal fuzzy white stripe) and tries to maximize profit by choosing the lowest possible bid that does not fall in a cell of the zero‐profit yielding upper right triangle. (b) Simulations enacting bidding behaviour of learning algorithms. Artificial bidders (left column: best‐fitting DL algorithm; right column: best‐fitting RL algorithm) were pitted against the subjects of the prerecorded dataset for 29 sessions and their preferred bids averaged within each trial and market type. (Upper left) Estimated prior parametric action‐value functions (using a Beta distribution with rescaled support and range) for each market type. (Lower left) Simulated maxima of each market action‐value function at each trial. (Upper right) Estimated initial preferred bids. (Lower right) Simulated preferred bids at each trial. Errorbars indicate s.e.m. [Colour figure can be viewed at http://wileyonlinelibrary.com]

The conditions differed in the number of sellers and buyers. In the *seller competition market* (SC), there were two sellers and one buyer (the subject); in the *no competition market* (NC), there were one seller and one buyer (the subject); and in the *buyer competition market* (BC), there were one seller and two buyers (one of them being the subject). In all market types, the outcome of the transaction was determined by pitting the highest buyer's bid against the lowest seller's ask price. If the former was strictly lower than the latter, then the transaction was not consummated, and the subject received the disagreement outcome: zero monetary units (MU). Otherwise, the subject received *10‐b* MU, where *b* is the bid of the subject. Hence, the win/loose structure was asymmetric: the win from an accepted bid was dependent on the bid amount, while the loss of fixed at 10 MU. We focused exclusively on buyer behaviour, unlike previous studies analysing all players’ behaviour (Grosskopf, 2003; Güth et al., 1982). The clearing price was set to be the maximum bid in order to study buyer behaviour specifically. In order to treat the task as a complete information game, we made the common assumption that all opponents assigned the same utility to the MU and to the fish. The task is a one‐shot game because opponents are assigned by repeated random matching. However, given that subjects play repeatedly in the three market types, this task also displays attributes of sequential games in the sense that what is being learned is not the type of one opponent, but the behaviour of a population of players as a whole. This topic has been previously explored from the viewpoint of strategic teaching (Camerer, Ho, & Chong, 2002). A colour‐coded buyer's payoff matrix representation of the NC game normal form is provided as illustration in Figure 2a (top centre).

The task can be formalized within the framework of Markov decision processes as a 4‐tuple (*S, A, R, T = p*(*s*_*i*_ *| s*_*j*_)*, R = p*(*r | s, a*)), denoting, respectively, the set of states consisting of the three market types *S=*{“SC”, “NC”, “BC”}*;* the set of actions consisting of all possible bids *A *=* *{0, 0.1, …, 10}*;* the state transitions probabilities, which are trivial because each market type evolves independently (*p*(*s*_*i*_ *| s*_*j*_)* = δ*_*ij*_, where *s*_*i*_ denotes market type *i* and *δ*_*ij*_ is the Kronecker delta); and the state‐action‐conditional reward probabilities *R = p*(*r | s, a*), which depend on the behaviour of the opponents such that *r = *10*−a* (where *a* is the action or bid) if the bid a overbids all opponent bids and ask prices depending on the market type, and *r = *0 otherwise.

Crucially, here the behaviour of the opponent is unknown a priori and can be assume to be internally represented as a probability distribution over competitor buyers’ bids and sellers’ ask prices. The form of this probability distribution is a decisive factor determining bidding behaviour, but under the modest assumption that subjects believe there exists a natural clearing price characteristic of each market with a reasonably small variance, we can model it approximately as a Gaussian distribution with centre at the estimated clearing price. For example, in a simple auction with one buyer and one seller, the buyer would hold an estimate of the (possibly varying) seller's ask price and would try to maximize profit by choosing the lowest possible bid that does not fall below the seller's ask price. Then, a strategy consisting of simply tracking competitor buyers’ bids and sellers’ ask prices would motivate a DL‐type and not RL‐type algorithms (see Figure 2a).

### 2.3 Task description

Subjects were informed that they were participating in a game investigating decision‐making. The game paradigm required buyers to fix their bids in advance. Their task was to buy fish on a market using a 10‐point Likert scale with increments of 0.1 MU. The initial position of the cursor on the Likert scale was randomized across trials. Collected fish led to a payoff: *p = *10−*b*, where *b* was the bid value in task MU, and 10 represented the maximum endowment the player could make use of in every transaction. Opponents were prerecorded human subjects replayed by a computer. In each trial, subjects played in one of the market types, which were looped throughout the experiment (24 blocks of 3 market types) in the order determined by a fixed sequence without repetition (of SC, NC and BC). One of the six possible sequences was pseudo‐randomly and independently assigned to each subject.

At the beginning of a trial, a MARKET stage (duration* = *5s, Figure 1a) informed subjects of the market type in the current trial. Next, a LOTTERY (duration* = *2s) stage consisted of a lottery determining whether subjects would be allowed to enter the market or not. In one of every six trials, subjects were not allowed to enter the market and had to move to the next trial. Otherwise, subjects entered the market and the CHOICE stage started. During the CHOICE stage (self‐paced, but with a prompt to answer quicker after 15s), subjects had to purchase (by bidding) fish in a market using a 101‐point slider scale. The feedback screen (OUTCOME, duration* = *6s) displayed the outcome of the transaction and the profit earned. In BC trials, when the competitor outbid the subject, that bid was made visible to the subject. Sellers’ ask prices were never disclosed. All inter‐stimulus intervals were jittered between 5s and 7s following a uniform distribution of duration 2s. The LOTTERY stage was included to assess the subject's differential neural response to being rejected from each market type. However, we found no differences in this respect.

Every subject played 24 trials of each market type (72 in total). The duration of each trial depended on the bid selection time and ranged from 21s to 61s, with an average of 39s. The total duration of the experiment was approximately 50 min.

The instructions explicitly informed subjects that they would play against prerecorded human players who had played the same game before against other human opponents. Our design precluded subjects from trying to manipulate their opponents’ behaviour in a sophisticated manner (Bhatt et al., 2010; Camerer et al., 2002). In each trial, the actions of the subjects’ opponents were matched according to the trial order of each market type (repeated random matching). Once inside the scanner but before the scanning started, subjects were trained on 6–10 trials, encompassing all market types (at least two trials of each market type). The training phase ended after subjects successfully and consistently manipulated the button box by placing their intended bid and then reported understanding the task.

After scanning, subjects were rewarded according to the following reward scheme (Roth et al., 1991): a fixed compensation of 300 Russian rubles (~5 USD) for participation, in addition to a bonus equal to the sum of the profit earned in three random trials multiplied by 15 MU (~5–12 USD in total).

The prerecorded data were recycled from a previous pilot study that implemented the same paradigm. Its design was identical to that of the present study with the following exceptions: 32 subjects played with real opponents in anonymous groups on desktop computers with conventional keyboards, and they played against each other, simultaneously, in the same room. The game was programmed in z‐Tree (Fischbacher, 2007). Subject roles were randomly assigned to buyer *or* seller throughout the duration of the experiment. Both seller and buyer had to set their respective ask prices and bids beforehand. The total number of trials amounted to 240 (40 periods with 6 rounds per period). In a post‐experiment check, we found that the behaviour of buyers in the prerecorded data was indistinguishable from the behaviour of buyers in the current participant cohort.

### 2.4 Stimulus presentation and response collection

The visual stimuli were projected with an LCD projector onto a rear screen. This screen was reflected by a mirror attached to the MRI head coil, subtending approximately 20 degrees of visual angle. The task was programmed using Presentation software (version 18.0, Neurobehavioral Systems). Responses were collected through three response buttons: the right thumb shifted the cursor to the right, the right index shifted it to the left and the left thumb confirmed bid choices.

### 2.5 Computational algorithms of adaptive learning

We implemented, fitted, tested and simulated six learning algorithms, including model‐free and model‐based RL and DL algorithms, with ad hoc parameters (Table 1). The data set consisted of the aggregated sequence of all trials played by the 27 subjects with the same prerecorded opponents. The important parameters were the learning rate (a measure of how much weight was given to recent feedback with respect to older feedback) and the randomness of choice, embodied in the inverse temperature of the softmax function (a measure of degree of action selection randomness) for RL algorithms, and in the dispersion parameters for DL algorithms. The dispersion parameters could be specific to the upper or lower side of the preferred bid and to the previous trial outcome contingency. The performance of the null algorithm, consisting of assigning uniform probability to all outcomes, was also computed as benchmark.

**Table 1:** Ranks and BIC scores for all fitted algorithms

Rank by BIC	Random effects (RFX)^a^		Fitted parameters	Fixed effects (FFX)			Agent name	Agent type	Number of parameters
	Negative log‐likelihood per subject	BIC per subject		Negative log‐likelihood per subject	BIC per subject	Fitted parameters			
1	28.04 ± 3.97	64.97 ± 11.50	*α* = 0.58 ± 0.04	34.15 ± 2.64	68.63 ± 5.41	*α* = 0.53	Leptokurtic DL with delta rule	Leptokurtic jitter + delta rule + DL	5
			*σ*_*a*_ = 0.61 ± 0.06			*σ*_*a*_ = 0.70			
			*σ*_*r*_ = 0.79 ± 0.06			*σ*_*r*_ = 0.79			
			*σ*_0_ = 0.56 ± 0.06			*σ*_0_ = 0.65			
			k = 0.30 ± 0.03			k = 0.39			
2	31.79 ± 2.08	67.14 ± 7.72	*α* = 0.56 ± 0.05	39.21 ± 2.55	78.55 ± 5.21	*α* = 0.38	Gaussian DL with delta rule	Gaussian jitter + delta rule + DL	2
			*σ* = 0.89 ± 0.07			*σ* = 1.09			
3	35.90 ± 1.89	78.91 ± 7.33	*n*_up_ = 0.31 ± 0.04	46.26 ± 2.10	92.78 ± 4.32	*n*_up_= 0.20	Leptokurtic naive DL	Leptokurtic jitter + DL	4
			*n*_down_ = 0.11 ± 0.02			*n*_down_ = 0.06			
			*σ*_*a*_ = 0.78 ± 0.05			*σ*_*a*_ = 1.06			
			*σ*_*r*_ = 0.82 ± 0.09			*σ*_*r*_ = 1.16			
4	96.19 ± 1.71	195.94 ± 6.98	*α* = 0.20 ± 0.05	101.98 ± 2.04	204.09 ± 4.21	*α* = 0.09	Model‐based counterfactual RL	Softmax + Counterfactual learning RL	2
			*β* = 1.37 ± 0.12			*β* = 1.00			
5	101.13 ± 1.00	205.82 ± 6.15	*α* = 0.002 ± 0.001	103.88 ± 1.55	207.89 ± 3.22	*α* = 0.00	Model‐free RL with coarse bid space	Softmax + model‐free RL	2
			*β* = 1.40 ± 0.11			*β* = 0.99			
6	100.65 ± 1.26	204.86 ± 6.07	*α* = 0.02 ± 0.01	103.45 ± 1.66	207.03 ± 3.44	*α* = 0.01	Model‐free RL	Softmax + model‐free RL	2
			*β* = 1.44 ± 0.14			*β* = 1.042			
7	120.26	242.30	–	120.26	242.30	–	Null model	Null	1

Note

“Jitter” refers to the shape of the probability distribution function used to model the variability of the bid selection process. *α*: learning rate; *β*: inverse temperature; *σ*_*a*_ *,σ*_*r*_ *,σ*_0_ : variance of Laplace distributions; *k*: proportion of trials with explorative (risky) versus exploitative (safe) bids; *n*_up,_ *n*_down_: fixed nudge size in the naive nudger algorithm. ±signify standard error of the mean across subjects.

Some instances of the RFX log‐likelihood optimization did not converge. Only those which achieved convergence are used.

In our task, there is only one state (each of the market types), unlike typical scenarios for RL agents, where the phase space comprises many states. The “native” action space consisted of 101 bid sizes. Although schemes for RL on continuous spaces have been proposed (Doya, 2000; Van Hasselt & Wiering, 2007), we opted to use a coarse “binned” representation of the native action space for our RL models, fitting multiple candidate algorithms informed by task‐specific assumptions. For the DL algorithms, we used the native action space.

To design the computational learning algorithms, based on preliminary data and heuristic reasoning, we devised a conceptual learning model of repeated bidding. The model requires at least three computational processes: (a) recognition of the different market types, (b) an internal representation of bid space and (c) model‐based learning optimizing bid choices.

#### 2.5.1 Model‐free RL

First, we modelled participants’ decisions using a Rescorla–Wagner (RW) like model‐free RL algorithm which learned to ascribe, maintain and update values attached to actions (Sutton & Barto, 1998). Here, the problem lies solely in choosing a single bid repeatedly. The basic action‐value updating equation was

$$Q_{m,t+1}\left(i\right)\leftarrow Q_{m,t}\left(i\right)+\alpha \left(r-Q_{m,t}\left(i\right)\right),$$

where *Q*_*m,t*_ (*i*) is the action‐value function with a value for each possible bid *i* given market type *m* at trial *t*, and *α* is the learning rate regulating the speed of action‐value updating. Action values were learned independently for each of the three market types. The policy for selecting a bid in each trial was a conventional softmax function,

$$P_{m}\left(i\right)=\frac{e^{\beta Q_{m}\left(i\right)}}{\sum_{j\in B}e^{\beta Q_{m}\left(j\right)}},$$

where *P*_*m*_(*i*) is the probability of choosing bid *i* in market type *m*,* β* is the inverse temperature parameter regulating the randomness in action selection, and *B* is the space of actions (bids). Clearly, such naive algorithm would perform very poorly given that it neglects the incentive structure of the game and the low ratio of samples (trials) to possible actions. Therefore, we binned the 101 actions into 11 uniform tiles (which speeds up learning), and we initialized the action‐value function distribution for each market type with a modified Beta distribution fit to the subject‐pooled first trial bids (Figure 2b, upper left). This furnishes efficient priors based on the subject's pregame beliefs about market types. Conventional Beta distributions are parameterized with two shape parameters and are defined on the real interval [0, 1], and their definite integral equals 1, but in our task the action‐value space spanned the interval [0, 10], and the sum of action values is not constrained. Thus, we rescaled both the support (from [0, 1] to [0, 10]) and the range of the Beta distribution to yield a usable prior for the *Q*_*m*_ functions.

#### 2.5.2 Model‐based RL with counterfactual learning

Other models are more suitable when relevant prior information is known about the task structure that can be crucial to solve complex tasks where model‐free RL becomes unwieldy. We used counterfactual learning, which can be regarded as an extension of model‐free RL where the value function is updated contingent not only on the currently chosen action feedback, but also on non‐chosen actions based on a model about the contingent rewards of foregone actions. This model is derived from the observation that in auctions, any bid lower than the ask price of the seller (and thus lower any previously accepted bid) would have been also accepted, had it been chosen. Value updating occurs for actions that were not chosen, but which are nevertheless updated based on the assumption that they would have been updated had they been chosen. Here, counterfactual learning is carried into effect explicitly as a model‐based RL algorithm which asymmetrically updates through the RW or delta rule the whole domain of bid choices every time a bid is selected, conditional on both the bid value and the feedback. Overall, it can be considered a hybrid of value function and model‐based algorithm. Although the RW and delta rule refer essentially to the same concept of gradient‐based incremental learning, from here we will use the more general designation delta rule because the name RW is historically associated exclusively to value‐based learning.

We applied the following rule sketch: for every bid *b* selected, if it is accepted (rejected), increase (decrease) the value of the action‐value function for all actions *i* which satisfy *i *> *b*. This, however, does not specify how much to decrease or increase the value of actions. We chose to update values conditioned on the outcome of the current transaction only for the higher or lower range of bids for accepted and rejected trials respectively, as follows.

If *b* accepted: for all *i < b*, $Q_{m,t+1}\left(i\right)\leftarrow Q_{m,t}\left(i\right)$

for all *i ≥ b*, $Q_{m,t+1}\left(i\right)\leftarrow Q_{m,t}\left(i\right)+\alpha \left(r-Q_{m,t}\left(i\right)\right)$

If *b* rejected: for all *i ≤ b*, $Q_{m,t+1}\left(i\right)\leftarrow Q_{m,t}\left(i\right)+\alpha \left(0-Q_{m,t}\left(i\right)\right)$

for all *i > b*, $Q_{m,t+1}\left(i\right)\leftarrow Q_{m,t}\left(i\right)$, where *α* is the learning rate, and *r*_*i*_ is the counterfactual reward, that is, the reward the player would have received had she selected the bid *i*. For the current trial bid *b*,* r*_*i*_ *= r*_*b*_ *= r*, the reward actually obtained. The action‐value function distribution was initialized for each market type with a Beta distribution fit to the pooled first trial bids.

#### 2.5.3 DL: a value‐free, model‐based learning algorithm

DL is a learning mechanism suggested for repeated games (Selten & Buchta, 1994). DL requires an a priori knowledge about the structure of the environment, and it is suitable only under specific circumstances: the space of feasible actions should be a totally ordered set (all its elements satisfy some mutual relationship by which they can be unambiguously characterized by a single index), and there should exist a unique optimal action associated to each game environment at each time point. Our task satisfies these conditions: bids are ordered respect to a magnitude of interest (bid size or bid value), and in each market scenario, there is a noisy clearing price whose average may or may not exhibit a time‐dependent drift. If the game environment is not stationary, DL will track the optimal price with some lag. The optimal action will depend in general on the utility functions of players (which comprises social preferences), and on the choice randomness of competitor buyers and sellers, but assuming that typical sellers (buyers) entertain reasonably stationary ask prices (bids), the optimal bid should be approximately the unique minimal bid below which all other bids are rejected.

The DL scheme is effectively a myopic policy that operates without the need of action‐value functions, by nudging the bids up or down depending on a directional signature (DS): whether the previous bid was accepted or rejected. This allows to model the payoff structure of choices around the optimal action, which is markedly asymmetric in our study because overbidding entails a reduction in the profit proportional to the overbid, but underbidding entails zero profit. The difference between RL and DL is apparently the small implementation detail of whether to cache actions or values, but it's a fundamental difference (Daw, Niv, & Dayan, 2005).

In every trial of market type *m*, DL is implemented by picking a bid from a unimodal probability distribution *P*_*m*_(*b*) centred in the preferred bid (the lowest accepted bid estimate). If the selected bid is accepted (rejected), then the preferred bid is increased (decreased). The preferred bid for the first trial of each market type was set to equal the mean of the pooled first trial bids. Unlike RL algorithms, DL algorithms lack the notion of expected value and therefore of RPE. In the DL algorithm, the variables tracking currently estimated action values are not conventional expected values, but rather, an estimation of the value of the maximum reward obtainable, namely the preferred bid value (PBV). Computing an expectation over a probability distribution of values associated with actions is not possible in a DL algorithm because there is no action‐value function over which a measure can be integrated, but PBVs can be interpreted as a rough equivalent of the conventional expected values of RL algorithms. Thus, it is possible to define a *pseudo‐RPE signal* as a RPE where the expected value is assumed to be the currently preferred bid.

This framework still leaves unspecified how much to decrease or increase the preferred bid, so we devised and fitted three adaptive learning algorithms based on DL.

#### 2.5.4 DL delta rule with Gaussian noise

This is perhaps the simplest conceivable DL model. We can update values conditioned on the outcome of the current trial by making the gain depend on the PBV and the reward received: $A_{m,t+1}\leftarrow A_{m,t}+\alpha \left(r-A_{m,t}\right)$, where *α* is a gain akin to the learning rate in RL, *A*_*m,t*_ is the preferred bid at trial *t*,* m* is the market type (SC, NC, or BC), and *r* is the reward. Here, the policy for bid selection accounts for noisy decision‐making by means of a Gaussian distribution function of bids around the preferred bid: $P_{m}\left(i\right)=\frac{1}{\sqrt{2\sigma^{2}\pi }}e^{\frac{-{\left(i-A_{m}\right)}^{2}}{2\sigma^{2}}}$, where *σ* is the standard deviation and *A*_*m*_, which is equal to the preferred bid for market type *m*, is the mean.

#### 2.5.5 Naive DL with asymmetric leptokurtic noise

This algorithm consists of simply “nudging” the bid up and down, but taking into account, the incentive structure of the game by doing it asymmetrically with respect to the two sides of the preferred bid. Contingent on the outcome of the transaction, the preferred bid is updated as follows:

If accepted, $A_{m,t+1}\;\leftarrow A_{m,t}+\;n_{\mathrm{up}}$, and if rejected,

We chose ad hoc a leptokurtic probability distribution function to model the noise around the preferred bid because it fits the data better than the Gaussian distribution (see Figure 1c). The distribution of bids (Figure 1c) is markedly asymmetric and non‐Gaussian, specifically with fatter tails and a thinner peak.

$P_{m}\left(i\right)=\frac{1}{2\sigma_{m}}e^{\frac{-\left|i-A_{m}\right|}{\sigma_{m}}}$ for *i > A*_*m*_ after previous trial rejection,

$P_{m}\left(i\right)=\frac{1}{2\sigma_{l}}e^{\frac{-|i-A_{m}|}{\sigma_{l}}}$ for *i < A*_*m*_ after previous trial acceptance, and for the rest of (rare) cases, where *P*_*m*_(*i*) is the Laplace distribution of bids *i* for market type *m*, and *σ*_*m*_, *σ*_*l*_ and *σ*_0_ are parameters proportional to the standard deviation of the (asymmetric) Laplace distribution. This is similar to the “RW(rew/pun)” algorithm of Guitart‐Masip et al. (2012), but with the important difference that here the updates occur in the action space instead of in the value space. This captures the intuition that the tail above the preferred bid after rejections is fatter than the tail below the preferred bid after acceptances.

#### 2.5.6 DL delta rule with asymmetric leptokurtic noise

**Figure 3:**
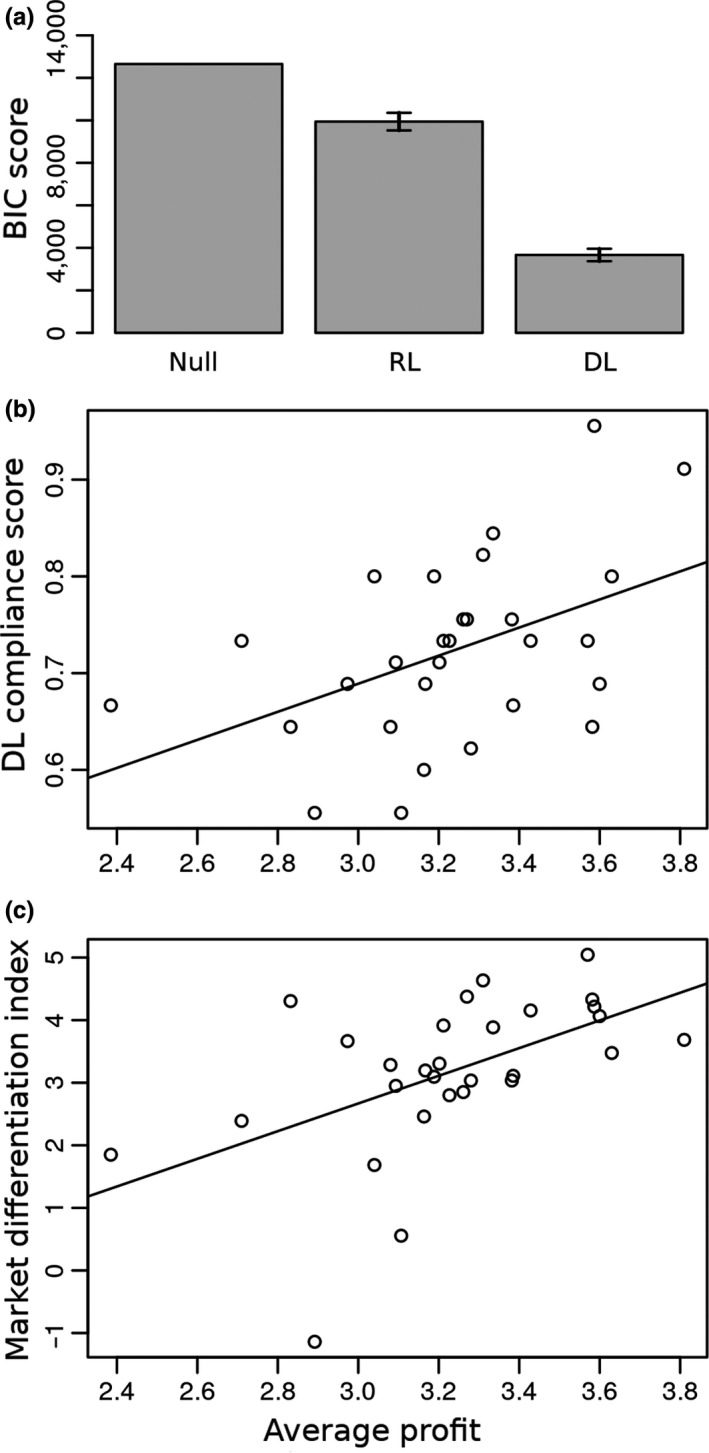
Algorithm fit scores and correlations with individual profits during the task. (a) BIC scores averaged within algorithm classes (DL: models 1‐3, RL: models 4‐6 in Table 1). Error bars indicate 95% confidence intervals. (b) Correlation of market differentiation index with profits averaged across the whole task. The line slope corresponds to a (Pearson's product‐moment) correlation coefficient of 0.524 (*p* = 0.003). (c) Scatter plot of subjects’ DL‐compliance scores and profits averaged across the whole task. The line slope corresponds to a correlation coefficient of 0.466 (*p* = 0.01). N = 27

This algorithm incorporates both the asymmetric leptokurtic policy distribution and the delta rule‐based updating of the preferred bid. This was the best‐fitting algorithm (Figure 3a, Table 1). It included an additional parameter *k* which accounted for a different proportion of trials with explorative (risky) versus exploitative (safe) bids.

### 2.6 Learning algorithms optimization and software

Following the usual approach in estimation problems with a small number of trials, a global objective function (the log‐likelihood of aggregated data) was optimized with yoked parameters (fixed effects) across all subjects for all learning algorithms (Daw, O'Doherty, Dayan, Seymour, & Dolan, 2006). This reduces parameter estimator variances at the cost of losing the ability to make between‐subject parameters comparisons by pooling together between‐subject and within‐subject variability, but this is deemed to have little impact in the quality of the algorithm simulation predictions (Grinband, Wager, Lindquist, Ferrera, & Hirsch, 2008), and more importantly, it eschews the inter‐subject variation among parameter estimates which results in a rescaling of regressors which leads to poor results at the group‐level in fMRI data analysis (Daw, Gershman, Seymour, Dayan, & Dolan, 2011). Given the scarcity of within‐subject samples and the jagged geometry of the resulting objective functions, and that the random and fixed‐effects analyses yielded largely consistent results (Table 1), we preferred this fixed effects comparison over the alternative of running the numerical optimizer for each subject individually in an objective function with multiple local extrema, which can lead to overfitting and bad performance of the numerical optimizer (but see Wilcox, 2005). For each algorithm agent, negative log‐likelihood functions were constructed by making the agent play all 27 of the subject sessions. The log‐likelihood function was

$$l_{\mu }\left(\theta_{\text{yoked}}|D\right)=\sum_{s=1}^{27}\sum_{n=1}^{24}\sum_{m=\text{SC, NC, BC}}\log\left(P_{\mu }\left(b_{\text{sn}}|\theta_{\text{yoked}},f_{\text{sn}},m\right)\right),$$

where *l*_μ_ is the log‐likelihood function for model *μ*,* θ*_yoked_ is the parameters vector of model μ (for example, for naive RL, *θ*_yoked* *_= (*α*,* β*)), and *P*_*μ*_ is the likelihood of model *μ* choosing a specific bid *b* given parameters *θ* and feedback *f*_sn_ in market type *m* for subject *s* and block number *n*. A numerical local search optimizer was then run on each of the negative log‐likelihood functions, and the found minima were used to recover the maximum likelihood parameter estimations. Bayesian information criterion (BIC) scores were derived from the negative log‐likelihood values (Table 1).

To check for consistency, we also performed separate optimization routines for each subject objective function: $l_{\mu ,s}\left(\theta_{s}|D_{s}\right)$, with individual free parameters *θ*_*s*_ for subject *s*. The scarcity of data samples prevented convergence in some subjects, but converged instances yielded consistent BIC scores and parameter fits (Table 1).

Because subjects have 101 possible actions and they play only 60 times in all three market types, convergence of the model‐free RL algorithms is troublesome when parameters are fitted individually, since values are updated sparsely and rarely, and often the game ends without sampling all possible states or actions. This is a problem for algorithm fitting, and in particular, estimating 101 initial action values depletes all useful degrees of freedom during optimization. Therefore, either we simplified the initial action values using a three‐parameter (as opposed to 101) density based on the Beta distribution (for RW‐type algorithms; Figure 2b, upper left) or we simply used the first round bids as initial conditions (for DL‐type algorithms; Figure 2b, upper right).

Data were processed with code written in Python with the scientific computing packages Numpy (RRID:SCR_008633), Scipy (RRID:SCR_008058), Matplotlib (RRID:SCR_008624) and Pandas. Purpose‐specific code was written to define the maximum likelihood functions used to estimate the parameters of the learning algorithms. The numerical optimizer employed was a bound‐constrained version of the Broyden–Fletcher–Goldfarb–Shanno algorithm, a local search technique which approximates local curvature. This algorithm is an implementation of a constrained optimizer of multivariate scalar functions belonging to the Python package Scipy. This optimizer was combined with a basin‐hopping heuristic (scipy.optimize.basinhopping) with at least ten “hops” to offset the probability that the optimizer would converge into a local minimum due to the jagged geometry of the log‐likelihood function.

### 2.7 fMRI data collection and analysis

#### 2.7.1 Data acquisition

The fMRI data were obtained using ascending interleaved slice acquisition with gradient echo T2*‐weighted echo‐planar imaging (EPI) sequence in a 3T Magnetom Verio equipped with a 32‐channel head coil (Siemens; Erlangen, Germany). Scanning protocol parameters were as follows: TE* = *30 ms; flip angle* = *80°; TR* = *2280 m; slice thickness* = *3 mm; no gap; slice matrix* = *64 × 64; number of axial slices* = *35; FoV* = *192 mm; and voxel resolution* = *3x3x3.7 mm.

High‐resolution structural MRI data acquisition used a T_1_‐weighted MP‐RAGE sequence. Parameters were as follows: TE* = *2.47 ms; flip angle* = *9°; TR* = *1900 ms; slice thickness* = *0.5 mm; slice matrix* = *512 × 512 × 176; number of slices = 176; FoV = 256 mm; and voxel resolution = 0.508 × 0.508 × 1 mm. These data were used for anatomical localization. A corrective routine aimed at counteracting susceptibility angled through the slice plane (z‐shimming) was performed by the scanner. The slice angle was tilted a negative 30° with respect to the anterior commissure–posterior commissure axis in the sagittal plane to reduce the unaccounted spatial components of the susceptibility gradients (Weiskopf, Hutton, Josephs, & Deichmann, 2006) and because this allows for better acquisition of the orbitofrontal cortex (Deichmann, Gottfried, Hutton, & Turner, 2003). The number of volumes acquired was on average 1,263, corresponding to a duration of approximately 48 min.

#### 2.7.2 Preprocessing

Images were processed using SPM12 (Wellcome Department of Imaging Neuroscience, Institute of Neurology, London, UK). Preprocessing of T2*‐weighted volumes consisted of rigid‐body model realignment within each session to a mean volume for head‐motion correction, unwarping of the residual variance using the field map, slice timing correction centred at TR/2, bias‐field correction, coregistration of T2*‐weighted volumes to the corresponding structural image (T1–weighted volume) and segmentation and spatial normalization to a standard T2*‐weighted template (Montreal Neurological Institute, MNI) for group analysis, spatial smoothing with an 8 mm Gaussian kernel and high‐pass temporal (128s) filtering. Fieldmaps were acquired using a dual echo 2D gradient echo sequence with echoes at 5.19 and 7.65 ms, and repetition time of 444 ms, and then used with the SPM FieldMap toolbox to correct EPIs for unwanted dropout due to variations in spatial magnetic susceptibility (Jezzard & Balaban, 1995; Weiskopf et al., 2006).

#### 2.7.3 GLM analysis

Eight event‐related regressors (delta sticks) were used to model the onset of the MARKET stage (MARKETxSC, MARKETxNC, MARKETxBC), LOTTERY outcome stage (for won and lost lotteries), CHOICE stage and OUTCOME stage (ACCEPTED and REJECTED). In addition, five parametrically modulated delta sticks were constructed: three for all stages of the task using the *preferred bid value* (PBV = 10‐PB): MARKET_PBV, LOTTERY_PBV, CHOICE_PBV; one for the pseudo‐RPE signal at outcome (OUTCOME_pseudo‐RPE) *based on the best‐fitting DL algorithm*; and one for the DS signal (OUTCOME_DS, consisting of + 1 for positive RPEs and ‐1 for negative RPEs). Both parametrically modulated and non‐modulated stimuli onset markers were convolved (first‐order expansion) with the canonical hemodynamic response function (HRF) implemented in SPM12 and entered into a general linear model (GLM). The motion parameters output from the preprocessing realignment routine were added to the design matrix as covariates to account for residual head‐motion effects.

In a separate analysis devoted to analysing the relationship between RPE and DS, two additional GLM regression matrices with three regressors each were constructed with the stimulus onset marker OUTCOME and the parametrically modulated regressors OUTCOME_DS and OUTCOME_RPE orthogonalized one respect to the other and vice versa (including other regressors irrelevant to learning processes did not change the results).

ROI activity in basal ganglia and PPC was extracted with the SPM extension MarsBar (Brett, Anton, Valabregue, & Poline, 2002). Masks consisted of 8‐mm spheres with centre in‐peak cluster of activity associated with PBV in PPC (MNI coordinates [+−47,−48,52]), and manually delineated anatomical subdivisions of basal ganglia were used as in Palminteri, Khamassi, Joffily, and Coricelli (2015), in both cases with also their contralateral homologues. Coefficient estimates (betas) were calculated by averaging over the coefficients of all voxels within their ROIs separately for each subject.

#### 2.7.4 fMRI statistics

Temporal serial correlations in fMRI data were removed using the residuals covariance matrix estimated by the restricted maximum likelihood routine in SPM12 to satisfy the sphericity assumption needed for doing inference (Starke & Ostwald, 2017). Subject‐level effects were fitted individually to their design matrices, and the resulting regression coefficients were taken to a random effects group‐level analysis, where the final coefficients values and statistics were calculated using the summary statistics trick (Holmes & Friston, 1998). All reported fMRI statistics come from the group level.

Most decision‐making studies model brain activity lasting less than 4 s with delta sticks, but studies have shown that this activity often lasts until the motor response (Grinband et al., 2008). Therefore, to ensure that such effects were not being ignored, we repeated the same analysis but with boxcar‐shaped regressors functions instead of delta sticks. We found no additional effects.

Activations of learning signals (DS and pseudo‐RPE) in the striatum and outside regions of interest (ROI) were reported at a voxel‐level threshold of *p* < 0.05 after voxel‐based family‐wise error rate (FWER) correction. Activations were reported in other ROIs and also in orthogonalized contrasts (i.e., the second parametric modulator regressor for a given event in the design matrix) when they exceeded a voxel‐level primary threshold of whole‐brain *p* < 0.001 uncorrected and a cluster‐level extent threshold of 10 voxels. Because such scheme yields a FWE‐corrected *p*‐value of 0.6–0.9 (Eklund, Nichols, & Knutsson, 2016), it was used only in regions that previous studies consistently reported to be involved in value‐based decision‐making and mentalizing in interactive play games (Barraclough et al., 2004; Bartra et al., 2013; Rilling, Sanfey, Aronson, Nystrom, & Cohen, 2004; Carter, Bowling, Reeck, & Huettel, 2012), in internal representation of the number line and manipulation of arithmetic objects (Dehaene, Molko, Cohen, & Wilson, 2004; Dehaene, Piazza, Pinel, & Cohen, 2003). These ROIs were orbitofrontal cortex, frontopolar and dorsolateral prefrontal cortex, anterior cingulate cortex, medial prefrontal cortex and temporo‐parietal junction. Cluster‐defining thresholds for all types of activity inference were appropriately set at *p *= 0.001 (Eklund et al., 2016; Flandin & Friston, 2019). Brain regions are displayed on a standard MNI template. All clusters from all figures are listed in Tables 2, 3 and 4. Thresholded cluster edges are indicated with black contour lines. Activation maps were dual‐coded (Allen, Erhardt, & Calhoun, 2012), where significance level and effect size were represented by means of colour saturation and hue, respectively, with MATLAB code from Zandbelt (2017).

**Table 2:** Neural activity related to market type recognition and expected value (Figure 4)

Contrast (Figure)	Region	Cluster p‐value FWE‐corrected	Cluster extent k	Peak T statistic	MNI (x, y, z)
MARKETxBC vs MARKETxNC (Figure 4a Left)	Left SPL	0.085	43	5.31	−33 −46 48
	Right SPL	0.044	53	4.55	36 −46 60
	Right ANG			3.92	39 −46 45
MARKETxSC vs MARKETxNC (Figure 4a Right)	Left SPL	0.818	9	3.75	−33 −52 48
CHOICE_PBV (Figure 4b)	Left SPL	0.630	15	3.99	−47 −48 52
REJECTED vs ACCEPTED, MDI‐modulated, group level (Figure 4c)	Right SFG	0.031	76	5.05	21 59 19
	Left SFG	0.125	47	4.53	−24 53 23
	Right MFC	0.582	17	4.46	6 29 −14
	Right ANG	0.301	30	4.26	60 −52 23
	Right TrIFG	0.258	33	4.18	54 32 4
	Left MSFG	0.528	19	4.11	−3 50 4

Note

Activity is thresholded at *p *< 0.001 (uncorrected for the whole brain), except for non‐orthogonalized contrasts in striatal areas, which are thresholded at FWER *p *< 0.05 voxelwise. x, y, z: stereotactic coordinates of the MNI template. Atlas labels were provided by Neuromorphometrics, Inc.

Abbreviations: AIns, anterior insula; ANG, angular gyrus; CblExt, cerebellum exterior; MFC, medial frontal cortex; MFG, middle frontal gyrus; MorG, medial orbital gyrus; MSFG, superior frontal gyrus medial segment; NAcc, accumbens area; OCP, occipital pole; SFG, superior frontal gyrus; SPL, superior parietal lobule; STG, superior temporal gyrus; TrIFG, triangular part of the inferior frontal gyrus.

**Table 3:** Neural activity coding error signals pseudo‐RPE and DS (Figure 5)

Contrast (Figure)	Region	Cluster p‐value FWE‐corrected	Cluster extent k	Peak T statistic	MNI (x, y, z)
DS (Figure 5a Left)	Left Putamen	<0.001	47	7.90	−30 −10 8
	Right CblExt	<0.001	147	7.70	33 −58 −40
	Left MorG	<0.001	20	7.68	−24 35 −18
	Right Putamen	<0.001	35	7.62	30 −10 4
	Left CblExt	<0.001	83	7.36	−15 −52 −18
	Left Caudate	<0.001	16	7.30	−24 −19 23
	Right Caudate	<0.001	51	7.29	24 −10 26
	Right Putamen			6.99	24 14 0
	Right CblExt	0.001	9	7.11	6 −70 −33
	Right OCP	0.001	12	7.02	18 −100 8
	Left Caudate	<0.001	13	6.46	−21 11 19
	Right SPL	0.003	6	6.38	45 −43 60
Pseudo−RPE (Figure 5a Centre Left)	Right CblExt	<0.001	119	8.49	18 −67 −22
	Left OCP	<0.001	25	7.26	−12 −103 4
	Right NAcc	<0.001	48	7.18	12 17 −11
	Right Putamen			7.16	21 14 −11
	Right Putamen	<0.001	14	6.87	30 −13 8
	Left SMG	0.003	7	6.81	−57 −34 45
	Left MFG	0.001	10	6.66	−36 35 30
	Left MFG	0.002	9	6.30	−39 38 15
	Right OCP	0.004	6	6.11	15 −100 11
	Left CblExt	0.003	7	6.09	−12 −52 −22
Ort‐pseudo‐RPE (Figure 5a Centre Right)	Left MFG	<0.001	197	5.14	−24 20 63
	Right SPL	0.315	29	4.65	27 −61 34
	Right MFG	0.196	38	4.63	42 14 56
	Left SPL	0.023	82	4.58	−21 −46 45
	Right SFG	0.283	31	4.31	27 14 63
	Right MFG	0.501	20	4.19	36 38 30
	Right MFG	0.924	5	4.09	48 41 26
	Right ACgG	0.728	12	4.03	12 38 11
	Left Nacc	0.609	16	4.01	−9 8 −7
	Left Caudate	0.788	10	3.89	−15 −4 23
	Right MFG	0.924	5	3.88	39 47 8
	Left ACgG	0.924	5	3.65	−3 32 −11
Ort‐DS (Figure 5a Right)	Left Caudate	0.070	56	5.36	−27 −7 26
	Left Putamen			4.43	−27 −10 8
	Right Caudate	0.227	34	5.06	24 −10 26
	Right Putamen			4.15	27 −10 11
	Right STG	0.057	60	4.87	57 −28 8
	Right Caudate	0.543	18	4.71	21 20 15

**Table 4:** Neural activity during OUTCOME stage associated with follow‐up bid increases (Figure 6)

Contrast (Figure)	Region	Cluster p‐value FWE‐corrected	Cluster extent k	Peak T statistic	MNI (x, y, z)
ACCEPTED bid increase‐modulated (Figure 6a)	Right Caudate	0.515	16	5.21	18 5 19
	Right Putamen	0.020	59	5.13	18 8 −11
	Right AIns			4.16	33 11 −18
	Left MFG	0.764	10	4.70	−33 56 19
	Left MFG	0.035	51	4.62	−30 41 34
	Right SMG	0.047	47	4.59	63 −34 19
	Left Putamen	0.202	28	4.50	−21 8 −7
	Right SFG	0.917	6	3.91	24 44 26
	Left MSFG	0.806	9	3.82	−9 50 0
REJECTED bid increase‐modulated (Figure 6b)	Right Putamen	0.818	9	4.19	24 14 −3

To localize potential brain regions involved in the computation of the economic transactions, we assessed on a trial‐by‐trial basis the correlations between neural data and model proxy variables. The data set comprising all the game sequences from all subjects was used to fit the parameters of each learning algorithm. The fitting process was informed by plausible assumptions about the players strategies, such as initializing prior bid values (see section “Computational algorithms of adaptive learning” for details). We selected the best algorithm based on BIC scores. Then, we derived time series of expected values (PBV) and prediction error (DS, pseudo‐RPE) signals from each of the learning algorithms by making each of the artificial bidding agents to enact human subjects behaviour. This entailed pitting the artificial bidders against the same sequences of stimuli that the human subjects played against, and in each trial computing the proxy variables (PBV, pseudo‐RPE, DS) furnished by their underlying learning algorithm, conditioned on the fact that they selected the same bids as the human subject they were enacting.

We standardized all algorithm proxy variables as z‐scores across subjects before entering them as parametric regressors in the design matrix. In the group‐level analysis, we used this analysis to link between‐subject differences to activations (Haruno et al., 2004).

Finally, a neural model comparison routine based on a SPM Bayesian model selection module was performed on anatomical ROIs encompassing striatum and inferior posterior parietal cortex. To assess the goodness of fit of both DL and RL algorithms to neural activity, we defined GLMs in OUTCOME, including either DS or RPE parametric modulators, respectively, and then estimated them using Bayesian statistics, which provided a measure of the evidence of the model for each subject. Log evidence was then fed to a BMS random effect analysis (Palminteri et al., 2015; Stephan, Penny, Daunizeau, Moran, & Friston, 2009), which computed the *exceedance probability* of each GLM within the anatomical mask.

## 3 RESULTS

3.1

#### 3.1.1 Behaviour across market types indicates heuristic (DL) learning of valuation

Overall, subjects successfully performed the double auction task under all types of social competition (72.47% of successful transactions). Transaction rates per market type were 92.44% (869/940) in SC, 74.68% (702/940) in NC and 50.26% (472/939) in BC market.

To estimate subjects’ beliefs about their human opponents and each market type prior to learning, we compared the bids in the first trial of each experimental session. On average, subjects bid 4.96, 5.13 and 6.55 monetary units (MUs) in the SC, NC and BC markets, respectively. A one‐way ANOVA test rejected the hypothesis that first mean bids were equal: *F*_2,137_ = 18.93, *p* = 6*10^‐8^. Thus, subjects discriminated among market types already before the beginning of the task. Mean reaction times (RT) were similar across market types (mean ± *SD*): 11.2 ± 3.6s, 11.1 ± 3.8s and 11.8 ± 3.8s for SC, NC and BC, respectively.

Next, we wanted to know how the bids and bid adjustments evolved over time and across markets. We tracked the evolution of subjects’ bid choices in each market (Figure 1b) by fitting a linear mixed‐effects model with random intercepts. Subjects gradually decreased bids in SC (beta = −0.027, *t*_588_ = −4.44, *p* = 5.4*10^‐6^) and increased bids in BC (beta = 0.086, *t*_587_ = 14.264, *p* = 4*10^‐40^), whereas in NC, we found a trend (beta = −0.009, *t*_588_ = −2.01, *p* = 0.045). Notice that the decreases in SC and increases in BC are not symmetric: subjects tended to increase the bids much more than decreasing them.

We reasoned that bid changes should depend directly on the subjects learning their success or failure in the previous bid they made. Hence, to inquire into the potential causes of bid evolution, we examined the effect of the previous trial outcome on the current bid. We tracked, on a trial‐by‐trial basis, the bid increments from one trial to the next within a given market type (Figure 1c). The distribution of these bid increments conditioned on the outcome of the previous trial displayed a skewed shape, with opposite skewness for the previous trial‐accept and previous trial‐reject bids. Such distribution can be roughly sketched as an asymmetric accept‐down/reject‐up rule or win‐stay/lose‐shift strategies (Nowak & Sigmund, 1994). Furthermore, the distributions of bid increments were qualitatively invariant across all market conditions, suggesting that the trial‐by‐trial learning rule underlying bid adjustments is independent of the market type. Therefore, we reasoned that the subjects’ market‐dependent bidding trends must be attributed largely to the opponents’ behaviour. This supports a view where subjects’ learning strategies (or algorithm) do not change among market types, yet, subjects explicitly recognize which market type they are in. This is indeed suggested by data in Figure 1b showing that the bids are rapidly rescaled between the different market types. We thus inquired what formal learning algorithm could best account for the learning behaviour and the evolution of bids (irrespective of the market type): conventional model‐free RL algorithms or model‐based algorithms that take into account the structure of the task (see below).

Finally, we examined whether subjects’ ability to bid successfully was related to how well they learned to identify the different market conditions. To get a coarse index of the degree to which subjects distinguished between the three market types, we devised the market discrimination index (MDI), calculated as the difference between the mean bid chosen over all trials for BC and SC conditions. Buyers who distinguished more market types, as assessed by the MDI, were more likely to receive higher profits (Figure 2b). Indeed, we found a correlation between profit earned and the MDI (*r* = 0.52, Pearson's product‐moment correlation, *t* = 3.20, df = 27, *p* = 0.003, 95% CI = [0.1955, 0.7473]). Thus, in our task, better market discrimination is associated on average with higher profit. Because in our task DL‐compliance score predicts profit precisely due to its ability to adapt quickly by caching preferred bids between market types, and thence finessing discriminability among market types, it should be as well correlated with MDI.

The above results gave us a hint that the observed behaviour may be accounted for by a DL algorithm of bid learning, where bids are nudged up or down depending on previous outcome. Importantly, DL requires a model of the “action (bid) space” to account for the directionality of bid adjustments. We also note that the traditional reinforcement learning schemes and DL differ in the learning signals they use to update decision‐making variables: a continuous reward prediction error (RPE) for RL and a binary error signal we denote by directional signature (DS) for DL (see Methods for details). In order to test our hunch that DL is used to learn bids in our task, we proceeded to test which DL or (and) RL algorithms could best explain the observed behaviour.

#### 3.1.2 Adaptive learning algorithm fits and model selection

We fitted six adaptive learning algorithms to the behavioural data. All DL algorithms fitted better than RL algorithms (Figure 2a), and RL algorithms failed to explain bid evolution in all market types. We believe this is to a great extent due to the lack in value‐based RL algorithms of the key prior knowledge underlying the structure of auctions: the asymmetric ordering of bid values around a preferred bid. Because of this, RL algorithms would require a large data set to learn action values to the point where they start being operationally useful. Since our subjects learned to bid successfully in the limited number of played trials, we argue that DL is the more efficient and appropriate learning strategy for our task.

Across all subjects, 74.99% (1586/2115) of the trials matched the behavioural predictions of the best DL algorithm. Conditioned on the outcome of the previous trial of the same market type, subjects behaved according to the DL algorithm in 76.26% (453/594) and 79.73% (1133/1421) of trials when their bids were rejected and accepted, respectively.

To visualize differences in predictive behaviour, we performed posterior predictive checks of the best‐fitting algorithms of RL type and DL type (Figure 2b), that is, we simulated replicated data under the fitted models and then compared these to the observed data (Gelman & Hill, 2006). This confirmed that DL‐type algorithms were able to learn rapidly profitable bids in each market type (Figure 2b, lower right), whereas RL‐type algorithms learned slowly, even when furnished with ad hoc rules to learn faster (as indicated by the maxima of action‐value functions; Figure 2b, lower left).

Importantly, subjects with a higher *DL‐compliance score* (the fraction of trials where they behaved according to DL) were more likely to receive higher profits (Figure 3c). We found a between‐subjects correlation between the profit earned and the proportion of trials compliant with DL (*r* = 0.47, Pearson's product‐moment, *t* = 2.74, *df* = 27, *p* = 0.011, 95% CI = [0.1204, 0.7113]). To confirm this, we took the best‐fitting DL and the best‐fitting RL models and simulated their bidding against the same prerecorded opponents as the subjects. Only the DL agent's bid evolution resembled the human one, with progressive increase in the SC bids and relative invariance of the NC and SC bids (not shown). Next, we proceeded to determine the neural underpinnings of repeated bidding learning.

#### 3.1.3 Fronto‐parietal cortical activity associated with recognition of the different market types

**Figure 4:**
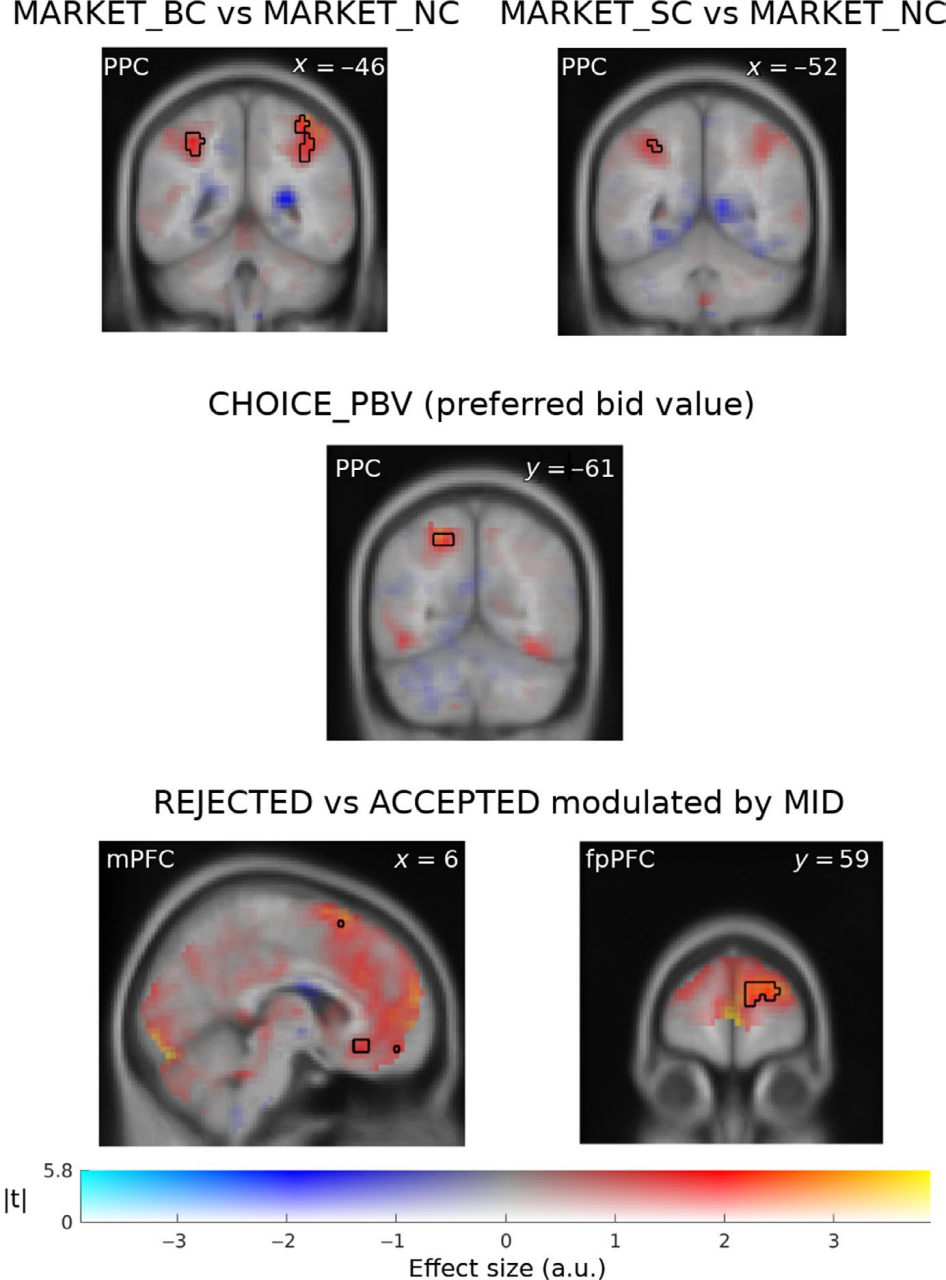
Neural activity related to market type recognition and expected value. (a) Left: stronger superior parietal cortex activity in BC as compared to NC condition during market entrance (MARKET_BC vs MARKET_NC). Right: stronger left superior parietal cortex activity in SC market as compared to NC market during market entrance (MARKET_SC vs MARKET_NC). (b) Activation reflecting modulation by the preferred bid during bid choice (CHOICE_PBV). (c) Feedback processing‐related activity (outcome stage, REJECTED vs ACCEPTED) modulated by individual differences in market differentiation index in the right medial frontal cortex (C Left) and frontopolar cortex (C Right). Activation maps are thresholded at p < 0.001 uncorrected, indicated by black contour lines. Clusters are listed in Table 2. Dual‐coded images represent both significance level and effect size by means of colour saturation and hue, respectively. [Colour figure can be viewed at http://wileyonlinelibrary.com]

To identify the brain regions associated with subjects recognizing the different market types, we analysed the neural activity during the MARKET stage of the task, which informs subjects about the market type at the beginning of each trial. We found that neural activity in the posterior parietal cortex (PPC) increased when subjects entered the competitive BC and SC markets (Figure 4a, Table 2) as compared to NC. The effect remained when the expected reward based on the preferred bid was regressed out, ruling out that it was a value‐related activation. The other pairwise subtraction contrasts between market types revealed no differences in activity.

To further investigate neural activity underlying the recognition of the different market types, we used the MDI as a covariate in the group‐level analysis. The between‐subject differences were manifested only in the prefrontal activity during processing of outcomes (OUTCOME stage, Figure 4b), specifically in a region bridging the bilateral medial frontal and superior frontal gyrus, adjacent to the frontopolar prefrontal cortex (fpPFC) and in mPFC (Figure 4c). Thus, fronto‐parietal activity was associated with the recognition of market types.

#### 3.1.4 Posterior parietal cortex activity associated with the internal representation of bid space

**Figure 5:**
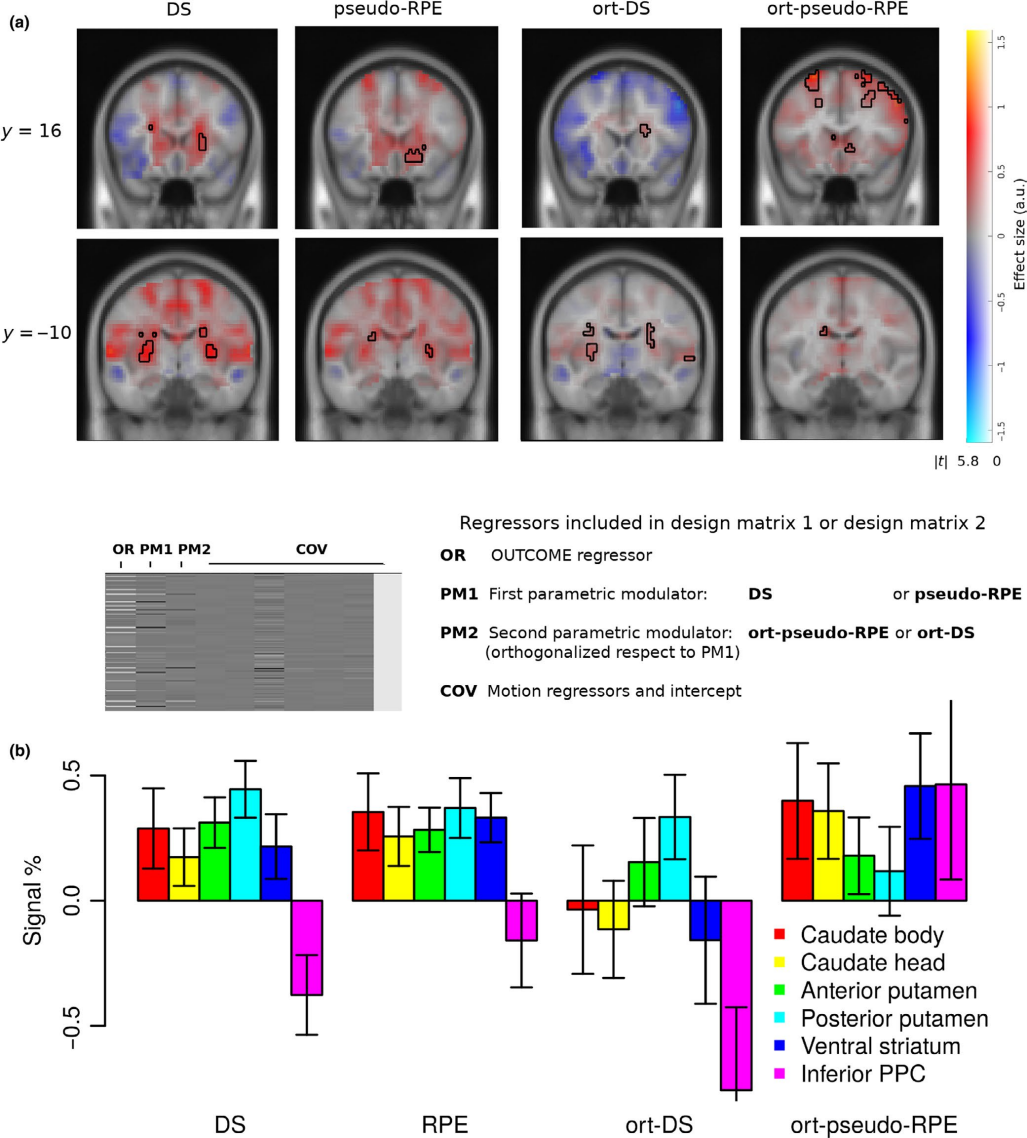
Neural correlates of pseudo‐RPE and DS signals based on the best‐fitting DL algorithm in anterior putamen and nucleus accumbens area and posterior putamen during OUTCOME. (a) Correlated activity in the anterior (y = 16) and posterior (y = −10) putamen was stronger for pseudo‐RPE and DS, respectively, during feedback. From left to right columns: pseudo‐RPE (*p *< 0.05, FWER), DS (p < 0.05, FWER), pseudo‐RPE orthogonalized with respect to DS (*p *< 0.001, unc) and DS orthogonalized with respect to pseudo‐RPE (*p *< 0.001, unc). The exemplary design matrix illustrates the correspondence between first and second parametric modulators and non‐orthogonalized and orthogonalized regressors, respectively. (b) Barchart of signal estimation (in grand mean percentage) by brain region. Signals were averaged within anatomical ROIs for basal ganglia (Palminteri et al., 2015) and on an 8‐mm sphere in PPC. oDS and oRPE correspond to DS and pseudo‐RPE signals after being orthogonalized with respect to each other, respectively. Activation maps DS and pseudo‐RPE are thresholded at *p *< 0.05 FWER‐corrected, whereas ort‐DS and ort‐pseudo‐RPE at *p *< 0.001 uncorrected. Clusters are listed in Table 3. [Colour figure can be viewed at http://wileyonlinelibrary.com]

To find brain areas whose activity encoded an internal representation of bid space, we used the preferred bids provided by the fitted DL algorithm as a covariate regressor at the CHOICE stage. We found activity modulation in the PPC (Figure 4b). This indicates that learned preferred bids are encoded in the PPC. Bids are real numbers, and their representation in the PPC is compatible with previous studies showing evidence for encoding of a number line in PPC (Dehaene et al., 2003). Moreover, the PPC region associated with the preferred bid value was also strongly modulated by both pseudo‐RPE and DS signals (Figure 5b).

#### 3.1.5 Striatal activity associated with trial‐by‐trial adaptive learning

In order to identify the neuronal representation of the learning algorithms used, we compared the explanatory power of RL and DL algorithms over the neural activity in the two areas most relevant to the task: striatum and PPC. We calculated the *exceedance probability* (Stephan et al., 2009) for each algorithm, given the brain imaging data gathered from all subjects. The exceedance probability was calculated using Bayesian model comparison of GLMs regressing the learning signals, DS for DL and pseudo‐RPE (the RPE based on the accepted preferred bids of the DL algorithm, see below) for RL. The analysis confirmed the explanatory power of the DL algorithm to be stronger than that of the RL algorithms: the P_exc_(DL) = 0.9533 > P_exc_(RL) = 0.0467. This yields a Bayes factor above 19, which indicates clearly strong evidence (Kass & Raftery, 1995) in favour of DL.

Therefore, we used the variables provided by the best‐fitting DL algorithm to search for neural correlates of the outcome evaluation and learning during the CHOICE and OUTCOME stages. In particular, we asked whether DL and RL neural learning signals could be distinguished. We reasoned that it is unsound to search for correlates of variables extracted from the ill‐fitting RL algorithms (e.g.,, their RPEs would be grounded on possibly very inaccurate expected values and thus be poor indicators of learning behaviour). Therefore, we instead compared RPE and DS signals by using the best‐fitting DL algorithm and calculating RPEs based on the reward expected from accepted preferred bids, which we refer to as pseudo‐RPE. We then performed a whole‐brain analysis for the OUTCOME stage and compared DS and pseudo‐RPE.

Neural correlates of both DS and pseudo‐RPE were found in the striatum (Figure 5). Because DS and pseudo‐RPE are highly correlated, we orthogonalized both regressors with respect to each other: *ort‐pseudo‐RPE* (*pseudo‐RPE orthogonalized with respect to DS*) and *ort‐DS* (*DS orthogonalized with respect to pseudo‐RPE*). Interestingly, *ort‐DS‐*related activity was found primarily in the posterior putamen, whereas *ort‐pseudo‐RPE* strongly modulated activity of the caudate and ventral striatum (Figure 5). This is in line with previous studies reporting that neurons in the caudate nucleus could play a role in transforming expected reward into a spatially selective behaviour (Gold, 2003; Kawagoe, Takikawa, & Hikosaka, 1998; Lauwereyns, Watanabe, Coe, & Hikosaka, 2002).

Our results indicate that both DS and RPE signals are encoded in the striatum, but in anatomically dissociated areas, anterior and ventral regions encode an RPE learning signal, whereas the dorsal and posterior regions encode a binary DS learning signal. We further explored averaged signals within anatomical ROIs. A two‐way ANOVA (regions: [posterior striatum, anterior striatum], learning signal: [ort‐DS, ort‐pseudo‐RPE]) yielded an interaction (*p* = 0.0012; *F* = 11.08, df = 1). Although both signals are represented concomitantly, computational algorithm fits suggest that DS is the predominant learning signal.

**Figure 6:**
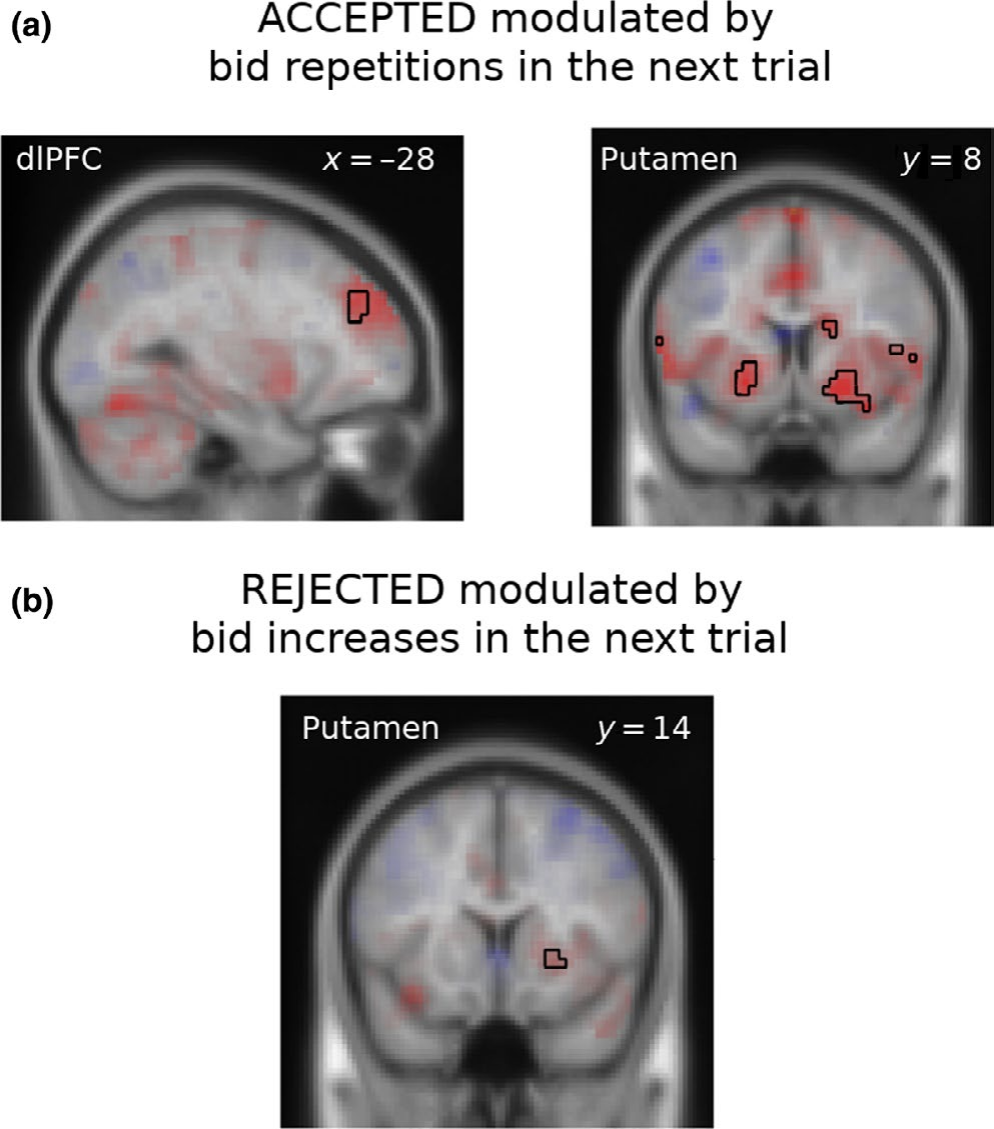
(a) Neural activity during positive feedback (ACCEPTED) in dlPFC (Left) and striatal (Right) areas that was modulated by bid increases in the next trial of the same market type. (b) Neural activity during negative feedback (REJECTED) in putamen that was modulated by bid increases in the next trial of the same market type. Clusters are listed in Table 4. [Colour figure can be viewed at http://wileyonlinelibrary.com]

Finally, we examined the relationship between learning‐related neural activity during OUTCOME and the behavioural adjustments. We computed a parametrical regressor modulated by the size of the subsequent adjustments of bids (the bid in the next trial of the same market type minus the bid in the current trial). Given that subjects after the *accepted* trials usually repeated or sometimes decreased their bids, the activity of the dorsolateral prefrontal cortex (dlPFC) and the ventral striatum in *accepted* trials was associated with subsequent bid repetition (Figure 6a). After the *rejected* trials, subjects most often increased or (less frequently) repeated the bid; activity of the right putamen during *rejected* trials was associated with subsequent bid increase (Figure 6b). Thus, neural activity in the dlPFC and striatum correlated with bid adjustments.

## 4 DISCUSSION

We investigated the neural underpinnings of learning to bid in double auctions. We found that buyers learned to choose bids using an effective decision‐making heuristic consisting of directional adjustments contingent on the previous trial outcome. As opposed to model‐free reinforcement learning, directional learning postulates the existence of a priori knowledge about the structure of the task. Namely, DL assumes that the action values of bids bear an order relationship; it and stores and updates the value of the preferred bid on an internal number line. Therefore, DL naturally fits market and auction decisions in which prices or quantities are the main strategic variables. Although one could object that DL and RL are intimately related, a crucial aspect distinguishes them: unlike RL, DL does not learn an explicit value function spanning all actions, but only a single preferred action.

Analysis of the first bids in each market type revealed that subjects discriminated among the market types already at the beginning of the game. Although subjects underestimated the effect of social competition in the different market types, they gradually learned to optimize their bidding decisions. Indeed, the learning curve for each market type exhibited an incomplete convergence towards the strict Nash equilibrium predicted for perfectly rational agents. Importantly, the fact that the RTs did not differ across the market types suggests that the differences of learning curves in three markets were not confounded by cognitive effort differences.

Since numerous fMRI studies have demonstrated neural correlates of RPE in the striatum (e.g., Haruno & Kawato, 2006; O'Doherty, Dayan, Friston, Critchley, & Dolan, 2003; van den Bos, Talwar, & McClure, 2013), we examined in detail pseudo‐RPE and DS‐related activity within this region. We found that the pseudo‐RPE signal was observed in the anterior and ventral striatal areas, whereas the DS signal was represented in the dorsal posterior striatal areas, particularly in the posterior putamen. According to the Bayesian model comparison analysis, the variability of the striatal activity was explained by DL better than by RL, supporting the pertinence of DL‐based bidding. This finding concurs with previous suggestions that neural learning signals in motivated decision‐making are not necessarily always RPE‐like (Behrens, Hunt, Woolrich, & Rushworth, 2008, supplement) and that a region of striatum is involved in learning stimulus–response associations and action selection (Jessup & O'Doherty, 2011). Although the coexistence of complementary yet exclusive value signals is not exceptional (Daw et al., 2011; Fouragnan, Queirazza, Retzler, Mullinger, & Philiastides, 2017; Lebreton, Jorge, Michel, Thirion, & Pessiglione, 2009), the reason underlying the concomitant DS and pseudo‐RPE signals in the striatum is unclear, since only DS explains the behaviour of participants. One possibility is that both learning systems operate concurrently, perhaps distributed over a broader network, as recent work that showed multiple distributed RPE valence and surprise representations (Fouragnan et al., 2017). In connection with this, it is interesting to note that the pseudo‐RPE signal orthogonalized w.r.t. the DS signal is conceptually analogous to an unsigned RPE (RPE “surprise”), that DS is analogous to RPE valence and that both signals were found to pertain to a common network for the computation of learning signals, in agreement with Fouragnan et al. (2017). It is also plausible that parallel computations could be adaptively deployed or left in standby by an arbitration process which decided which of them controls behaviour (Collins & Koechlin, 2012; Daw et al., 2011). Although these learning signals are difficult to decorrelate, a follow‐up study could clarify their relationship, in particular, whether these signals could be partially ancillary to bidding behaviour and be part of a hybrid DL‐RPE architecture.

Intriguingly, we also found that feedback processing‐related neural activity was parametrically modulated by the degree of bid adjustment in the next trial in dlPFC and striatum: activity in both regions was associated with bid increase or repetition in the next trial *regardless* of whether the bid was previously accepted or rejected (Figure 6a). We may posit that activity of the dlPFC subserves a cognitive control mechanism for tracking the preferred bid, and concomitantly striatal activity has a role in increasing the value of the currently preferred bid. This parallels the previously reported role of the dorsal striatum in updating action values (Balleine, Delgado, & Hikosaka, 2007; Haruno et al., 2004; Lauwereyns et al., 2002; Palminteri et al., 2012) and the parametric working memory encoding in the PFC reported by Romo, Brody, Hernández, and Lemus (1999). Activity predicting bid adjustments after rejection was also present in the putamen when subjects’ bids were rejected. To account for the role of the striatum in updating bids instead of values, we speculate that because the task revolves consistently around the bid choice, the reference magnitude for updating values was not the expected reward, but the preferred bid, as suggested by the best‐fitting DL algorithm. Although to our knowledge, such function has not been attributed to the striatum in previous studies, it is plausible that at least some neuronal submodules could compute bids instead of expected rewards because in our task, the bid is the natural operational variable (bid size is the only quantity that needs to be tracked) and is perfectly anti‐correlated with reward when accepted. The activity consistently associated with “nudging up” bids, and a similar signal reported in the superior PPC (Figure 4b) lends support to this hypothesis.

The DL‐type learning strategy requires a representation of an internal number line where the preferred bids are stored and actively updated. Our results indicate that this representation is implemented in the PPC (Figure 4a). Accordingly, Gläscher, Daw, Dayan, and O'Doherty (2010) also found neural signatures of model‐based prediction errors analogous to DS in the PPC in a Markov decision task, and the superior PPC has been implicated in directing spatial attention to a representation of an internal number line (Hubbard, Piazza, Pinel, & Dehaene, 2005). Moreover, we found activity associated with the preferred bid size in the left superior PPC, which has been also found to represent the relative value or probability of different actions (Sugrue, Corrado, & Newsome, 2005). Thus, during bidding, activity of the superior PPC could not only modulate attention to the internal number line, but also contribute to decision‐making. Other neuroimaging studies show that the activities of the superior PPC contribute to working memory (Koenigs, Barbey, Postle, & Grafman, 2009), arithmetic facts (Dehaene et al., 2004; Pesenti, Thioux, Seron, & De Volder, 2000) and quick value‐based decision‐making (Jocham et al., 2014). It is also interesting to note that a mechanism affording the representation of the preferred bid should be very similar to the neural integrators that have been proposed for explaining oculomotor control (Seung, 1998). Altogether, the superior PPC could participate in a calculation and representation of the preferred bid that is transmitted to motor areas to execute appropriate motor commands.

The ability to recognize market types is also critical for successful bidding. At the beginning of each trial, activity in the bilateral superior PPC was stronger in trials with higher social competition (SC and BC; Figure 4a). This activation could reflect neural activity monitoring the competitiveness in the current trial or retrieving relevant information (Vilberg & Rugg, 2008) about the current market type (i.e., the preferred bid). Activity in the superior PPC has been previously implicated in the processing of numerical information needed for the forthcoming motor selection (Sawamura, Shima, & Tanji, 2002). Thus, the PPC could set bargaining decisions into the appropriate social competition context by associating the specific market type with its associated DL‐learned preferred bid. Therefore, successful bidding could be subserved by the same computational processes underlying simple arithmetical calculations (Dehaene et al., 2004) and distance estimation. Between‐subject differences associated with the ability to distinguish the different market types in our study affected the activity of the fpPFC and vmPFC. This might indicate that subjects who distinguished better among market types, besides earning more profits, exhibited stronger activation of the higher‐order cognitive prefrontal areas associated with the appraisal of suitable models of the environment (Boorman, Behrens, Woolrich, & Rushworth, 2009) and mentalizing (Coricelli & Nagel, 2009; Hampton, Bossaerts, & O'Doherty, 2008). Congruently with previous fMRI studies, fpPFC activity might be involved in appraising the behaviour of opponents (Koechlin & Hyafil, 2007), whereas vmPFC activity might be involved in appraising the subject's own valuation during feedback.

In this study, we used prerecorded opponent data, which could affect behaviour through social preferences (van den Bos et al., 2008) and arguably may not allow us to disentangle precise market‐based prior strategies from feedback‐based learning. Although studies using live opponents (e.g., Carter et al., 2012) eschew this limitation, they cannot control well for variability induced by repeated mutual feedback, which was necessary in our study to control the bid variability in each market type. Further studies are needed to verify the role of feedback‐based learning in double auctions.

In conclusion, while the buyers were bidding under different levels of supply and demand, their behaviour was explained best by a simple learning heuristic. Between‐subjects higher compliance with DL predicted higher payoffs. Our results suggest that the PPC encodes an internal representation of a bid space that serves as a model on top of which subjects adjust and select bids, and posterior striatal activity was associated with a simplified learning signal characterized by a binary learning signal. Individual differences during feedback associated with activity in the dlPFC and superior PPC indicate the critical role of at least a rudimentary prior knowledge of the structure of the task and the differences among market types. In summary, we suggest that a learning heuristic based on a binary learning signal distinct from the conventional RPE signal solves the problem of repeated bidding in double auctions. Showing the learning mechanisms underlying bidding under social competition, this study paves new pathways for the discovery of neural mechanisms engaged in competitive, dynamic, complex decisions.

## COMPETING INTERESTS

The authors declare no conflicting financial interests.

## AUTHOR CONTRIBUTIONS

M.M. gathered data, wrote stimulus presentation code, analysed data, designed and fitted the learning algorithms, and wrote the manuscript. R.K and M.P. assisted in the collection of data. A.S. and B.G. supervised the study. V.K. designed the task, supervised the study and edited the manuscript.

## Data Availability

Source code implementing artificial bidders of DL‐ and RL‐type, model fits, and simulation results are available under the MIT licence, and they are freely downloadable from the web on the hosting service GitHub (https://github.com/mmartinezsaito/action-in-auctions). Functional imaging data and subjects’ behaviour logs are available in BIDS format (Gorgolewski et al., 2016) on the OpenNeuro database under a Creative Commons CC0 licence (https://openneuro.org/datasets/ds001966).
