# Supplementary figures and images for: Action in auctions: neural and computational mechanisms of bidding behaviour
Source: Eur J Neurosci. 2019 Jul 29;50(8):3327–48. doi: 10.1111/ejn.14492 (PMC6899836; doi:10.1111/ejn.14492)

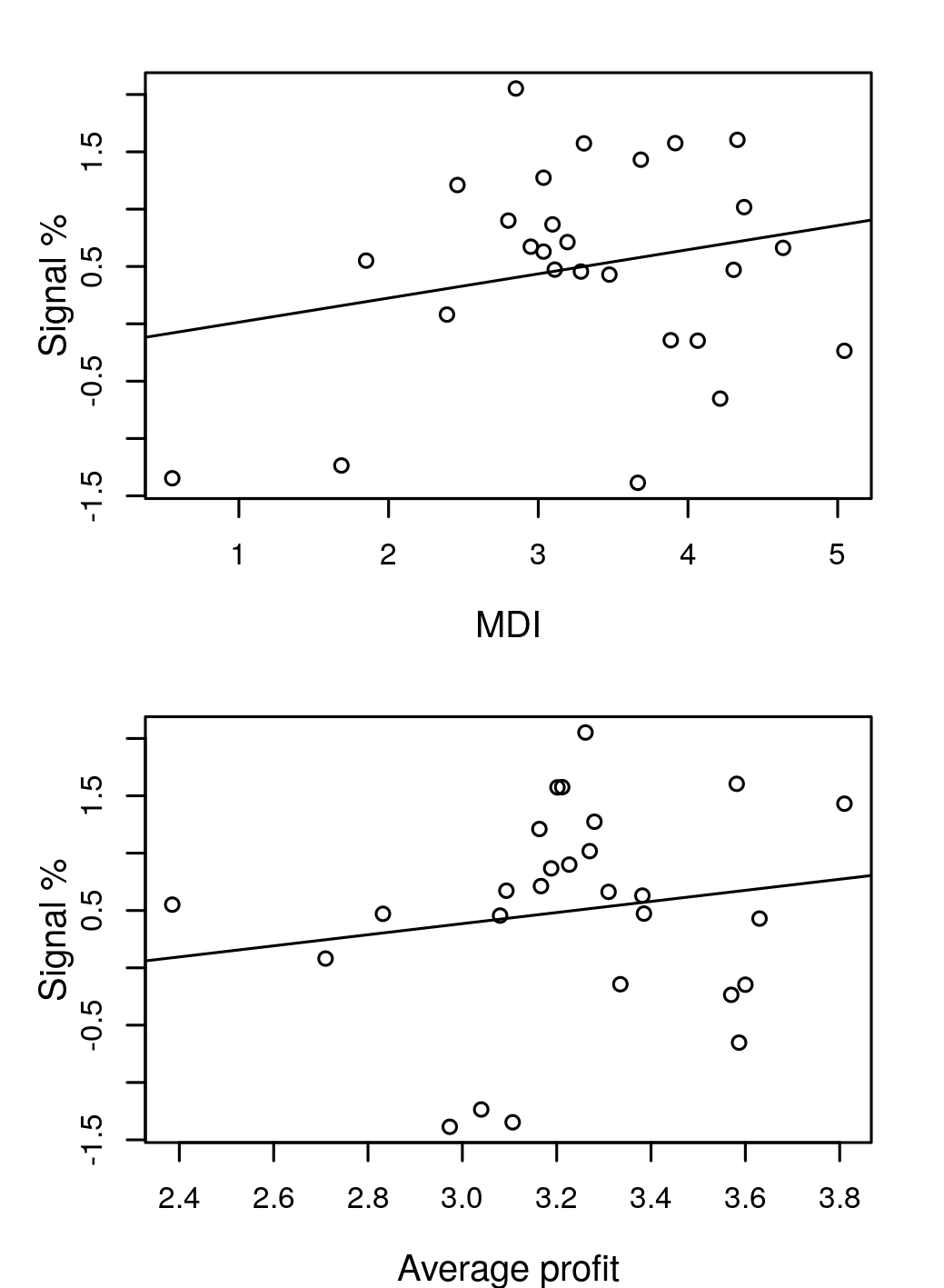

Supplement: Supplementary file 2 [file EJN-50-3327-s002.tif]
