# Supplementary material for: Action in auctions: neural and computational mechanisms of bidding behaviour
Source: Eur J Neurosci. 2019 Jul 29;50(8):3327–48. doi: 10.1111/ejn.14492 (PMC6899836; doi:10.1111/ejn.14492)
Supplement: Supplementary file 1 [file EJN-50-3327-s001.pdf]

EJN European Journal of Neuroscience FENS Federation of European Neuroscience Societies

Action in auctions: neural and computational mechanisms of bidding behavior

Martinez-Saito, Mario (contact); Konovalov, Rodion; Piradov, Michael; Shestakova, Anna; Gutkin, Boris; Klucharev, Vasily

---

Review timeline:

Submission date: 15-Nov-2018
Editorial Decision: Major Revision
Revision Received: 01-May-2019
Accepted: 24-May-2019

Editor: Guillaume Rousselet
Reviewer 1: Elsa Fouragnan
Reviewer 2: Dirk Ostwald

1st Editorial Decision 21-Jan-2019

Dear Dr Martinez-Saito,

We have now received comments on your article from two experts. They are both overall positive and have mostly requested clarifications and extra illustrations. In addition to the very constructive reviewers' comments, we request that you take the following comments into account.

You need to limit your conclusions to what was measured and you should phrase the presentation of the results to avoid the classic 'absence of evidence is not evidence of absence' error, for instance in this sentence:

"Reaction times (RT) did not differ significantly across market types: 11.2±3.6s, 11.1±3.8s, and 11.8±3.8s for SC, NC, and BC, respectively."

You can see examples of phrasing for frequentist results here:

https://discourse.datamethods.org/t/language-for-communicating-frequentist-results-about-treatment-effects/934

https://peerj.com/preprints/26857/

You could simply mention that the (mean?) RT were similar, and report the estimates, confidence intervals and p values, without dichotomising the results. Please also describe the measure of central tendency used for reaction times.

If you dichotomise p values based on an arbitrary threshold, then there is no such thing as "weakly significant". However, it is fine to treat p values as continuous measures of compatibility between the data and the model.

"we found a significant correlation" - we encourage you to remove the terms significant / non-significant as they do not add any information, they only provide the illusion of certainty.

All correlations should be illustrated using scatterplots.

Figure 1B: please add plots of the pairwise differences in another panel, so that readers can get assess effect sizes.

Figures 2A 4B: please use scatterplots instead of bar graphs.

Figures 3, 4A, 5: please provide non-thresholded maps. Here are examples and code:
Allen, E.A., Erhardt, E.B. & Calhoun, V.D. (2012) Data visualization in the neurosciences: overcoming the curse of dimensionality. Neuron, 74, 603-608.
mialab.mrn.org/datavis/
https://github.com/bramzandbelt/slice_display
https://github.com/spinicist/nanslice

Also, and this is an important issue, we strongly encourage you to post your data online (using figshare for instance) or to indicate in the article why you cannot share them. Please also provide your analysis code.

Please also attend to the following issues identified by our editorial office:
1. Separate figure legends will be needed
2. Please ensure that you provide a text and a figure file for the Graphical Abstract (as detailed in the instructions below).
3. You will need to provide more detailed information regarding the ethical committee that approved the work
4. Your bibliography must be carefully checked and put into EJN style at this stage

When revising the manuscript, please embolden or underline major changes to the text so they are easily identifiable and DO NOT leave 'track change' formatting marks in your paper. When carrying out your revisions please refer to the checklist below and visit the EJN author guidelines at www.ejneuroscience.org

When finalized, please upload your complete revised manuscript onto the website, as a Word file (.doc, or .docx). Please also ensure that a complete set of tables and figures is included as separate files, even if these have not changed from the originals. At this stage it is necessary to provide high resolution figures. Please see important instructions below.

Please go into https://mc.manuscriptcentral.com/ejn - Author Centre - manuscripts with decisions where you will find a 'create a revision' link under 'actions'. We ask that you please indicate the way in which you have responded to the points raised by the Editors and Reviewers in a letter. Please upload this response letter as a separate Word (.doc or PDF) file using the file designation "Authors' Response to Reviewers" when uploading your manuscript files. Please DO NOT submit your revised manuscript as a new one. Also, please note that only the Author who submitted the original version of the manuscript should submit a revised version.

If you are able to respond fully to the points raised, we would be pleased to receive a revision of your paper within 12 weeks.

Thank you for submitting your work to EJN.

Kind regards,

John Foxe & Paul Bolam
co-Editors in Chief, EJN

Reviews:

Reviewer: 1

Comments to the Author
Martinez-Salto et al. present an interesting study examining the neural correlates of biding strategies under different types of context varying in numbers of sellers and buyers. The authors propose to use a new model - the directional learning model - to describe behavior, which ultimately seems to do a better job at explaining participants responses than more classical model-free or model-based RL models. Their results indicate a complementary job of the RL-like signal (RPE) and the DL learning signal in the striatum which is interesting while others areas seem to mediate early strategies. The analyses are comprehensive and the results appear robust and carefully described. However, I think there remains some minor work to do to provide clarity for the reader on the key question that the paper is seeking to answer.

1) The BICs on the model fit are quite convincing (Figure 2a). The best DL model is shown to fit the average profit score in Fig 2c but this is a coarse data feature, and from the data it is not clear what differentiate the best DL from the others and the model-based models. Is it possible to visualize the data feature(s) that distinguishes the model predictions? E.g. presumably the models make different predictions as a function of the elapsed time since the beginning of a market type? Seeing as the critical imaging findings rest on model-derived value regressors, the conclusion that the DL model is the best model of behaviour needs to be shored up by additional analysis beyond a comparison of BIC scores.
2) Similarly, since the DL model is relatively unknown in the field and could be of interest for a larger audience, is it possible to have an intuition in a graph of its core features?
3) Finally, what is the relationship between the pseudo-RPE and the RPE derived from the other models? Would these correlate?
4) What's the relationship between the market discrimination index (MDI) and the DL compliance scores? It looks like they might be related from Fig. 2B&C.
5) The figures lack consistency and clarity in presentation. The task on figure 1A is hard to read while B&C others present different font size for similar elements. What is the error bar on the BIC score on figure 2A? In figure 3, the titles have a very low resolution. And what is the Z or T score for the statistical maps?
6) Why did the authors decide to remove 5 out of 8 regressors in their model when doing the orthogonalization of OUTCOME_DS and OUTCOME_RPE? Do the results remain unchanged if they use the exact same model (aside the orthogonalization)?
7) Would it be possible to see - as an illustration (scatter plot) - the correlation between the between-subject differences in MDI and the activity in the prefrontal cortex during processing of outcomes (OUTCOME stage, Figure 3B)? I find it unclear from the figure to see if it is a positive or negative correlation. What about the average profit score? Does that also correlate?
8) I am unsure whether the OUTCOME_DS is only the valence of OUTCOME_pseudo_RPE (+1 positive RPE and -1, negative RPE). It looks like it is from the methods. Is that right? In this case, orthogonalizing the two (OUTCOME_pseudo_RPE with respect to OUTCOME_DS) ends up creating almost an unsigned RPE and keeping the valence DS regressor intact. An unsigned ROE is then pretty much a surprise (how far from

EJN European Journal of Neuroscience FENS Federation of European Neuroscience Societies

expectation) signal. It would be interesting to discuss the results in light of recent work showing multiple valence and surprise representation in the striatum (Fouragnan et al., 2017, figure 5).

Reviewer: 2

Comments to the Author
Martinez-Saito and co-authors report on an ambitious computational cognitive neuroscience study into the neural correlates of sequential bidding behaviour. The manuscript is very well written, the methodology used adheres to the highest standards in the field, and the evaluation of the behavioural and neuroimaging data is extensive. In brief, the authors find that directional learning (DL) models outperform conventional Rescorla-Wagner-type reinforcement learning models in their ability to describe human choice behaviour in sequential bidding. Moreover, the authors identified correlates of DL model trial-by-trial parameters in the striatum, evidence for the encoding of the action space in the posterior parietal cortex, and a correlation of bid adjustment with neural activity in the DLPFC. I have a number of suggestions to further improve the clarity of this manuscript, which the authors may consider for a minor revision of their work.
Task description
I think that the choice environment could be characterized in a more formal and more quantitatively explicit manner. Specifically, participants performed the double auction task in three different conditions (seller competition, no competition, buyer competition) against the responses of pre-recorded human subjects replayed by a computer. I would think that the adaptive bidding behaviour of the participants can be appreciated more comprehensively, if the readers had information about these pre-recorded choices. For example, were the pre-recorded bids quantitatively similar to the data observed for the current participant cohort? Ideally, and because the authors already refer to concepts from the theory of Markov decision processes on occasion, the authors would describe their task by a tuple (S,A,R,p(s_t|s_{t-1}, p(r_t|s_t))) of state, action, and reward spaces, as well as state transition and state-conditional reward probabilities, respectively, with which the participants either interacted objectively (i.e., from the perspective of the authors) or subjectively (i.e., based on the cognitive modelling assumptions of the authors). This would allow readers to easier understand the task and appreciate the (in)appropriateness of certain choice policies used in the behavioural models.
Behavioural modelling
Model-free RL: I think the value Q_t(b|m) for the state-action-value function Q_t : S \times A -> \mathbb{R} would better be denoted by Q_t(b,m), as the vertical line is easily confused with probabilistic conditioning, which is not meant here. The same holds for the action probability P(b|m), where the potential confusion is even more severe, as a probability is denoted.
The authors report that they initialized the state-action-value function with a Beta distribution based on the subject-pooled first trial bids. This is somewhat confusing. A Beta distribution is defined on the interval [0,1]. The action space, on the other hand, appears to have consisted of the discrete set A = {0,0.1,0.2,…,10} with cardinality 101. Do the authors mean that they scaled the action space into the continuous interval [0,1] or that they used a multinoulli distribution over the discrete action space? It would also be helpful to visualize the initial and final state-action-value functions to appreciate the degree of adaptation throughout the task.
Model-based RL with counterfactual learning: the action conditional update rule on p. 12 is slightly confusing. First, I am not clear on the domain of the state-action value-function used here, with respect to the state-action-value function used for the model-free RL. In the current setting, the

function has three arguments, i
DL as value-free, model-based learning: The authors note that DL is suitable if there exists a unique optimal action. It may be helpful to mention the form of this unique optimal action for the current choice environment or at least provide a reference for its formal proof of existence. Naively, I would assume the optimal actions in the different markets scenarios are different, but exist, and depend on the expected bids of competitors, expected seller's price, as well as the specified loss and disagreement outcomes.
I think a figure that conveys the central assumptions of each behavioural model and provides some exemplary learning trajectories could increase appreciation of the different model types.
I strongly encourage the authors to upload their behavioural modelling code as well as the aggregated anonymized behavioural data to a data sharing platform such as the OSF or Github. As a standard for documenting the data, the BIDS format for behavioural data may be suitable.
Functional neuroimaging data
I appreciate that the authors attempted to estimate the power of their study, but I think the approach used (Cheng and Schwarzman, 2015) does not really relate to the statistical tests reported later, as these would have to be performed with the same statistical test implied by Cheng and Schwarzman (for a detailed review of the SPM p-values relied on in the current study, see e.g. https://arxiv.org/abs/1808.04075). I would suggest omitting the power calculation.
In the GLM analysis, the authors introduce the preferred bid value (PBV). I think it would help to introduce this central variable for the model-based analysis of the fMRI data already in the discussion of the DL model to make the link between the behavioural and functional imaging models more explicit.
I would suggest to supplement the GLM-based results figures with a depiction of an annotated exemplary design matrix, as this is the easiest way to communicate the designs used.
The sentence "Temporal serial correlations in fMRI data were removed with the (SPM12) autoregressive AR(1) model to satisfy the parameter estimation routine (ReML) assumptions" is askew. SPM12's ReML routine uses a covariance component estimation approach to estimate the degree of serial correlations. The estimated covariance matrix is then used to "whiten" the data, i.e. to remove serial correlations. The aim of this is to have the whitened data conform to the sphericity assumption necessary for the use of standard z, T, and F distribution in parametric inference (see e.g. https://doi.org/10.3389/fnins.2017.00504).
Unfortunately, the description of the analysis involving fitting fMRI data to the learning algorithm (p. 19-20, Results p. 24) is opaque to me. Which fMRI data features does this refer to? The trialby-trial nature of the description of the analysis seems to imply a trial-wise feature, but the extraction of this feature is not explicitly mentioned. This part of the manuscript should be improved significantly.
Table 2 and its caption are somewhat confusing. First, why do the authors report only z-scores, not T-values? The uncorrected peak voxel p-value reported in the penultimate column is commonly evaluated based on the respective test statistic, which would be a T-value in the current case (the cluster extent p-value is applied to T-values under the assumption of high degrees of freedom, in this sense, reporting the Gaussianized z-scores makes sense, although I think that most researchers, including myself, commonly assume that their study degrees of freedom are high enough, which in the current study with $n = 27$ is also the case). Second, the authors write that here and below all voxels are significant at least at $p < 0.001$, uncorrected for the whole brain. Does this refer to the cluster-defining threshold? From the methods part my understanding was that the cluster-defining threshold was set to $p < 0.05$ FWE corrected. Again, with respect to the penultimate column of the table: is this the corrected or uncorrected peak-voxel p-value? If it is

the uncorrected p-value, it would be redundant, because the entire map is thresholded at $p < 0.001$ according to the caption. Finally, with respect to the statement "cluster-level inference alone may yield inflated false positives (Eklund, 2016, Flandin and Friston 2017)" - I think the bottom line of both studies cited is that for an appropriately high cluster-forming threshold (e.g. corresponding to an uncorrected p-value < 0.001), the FWE corrected p-value for cluster extent is approximately correct for the resting state fMRI data analysed in these studies.
Finally, I would also encourage the authors to share the functional neuroimaging data in a standardized format, either openly, if possible based on ethical constraints, or in a protected format using data use agreements.

Authors' Response 01-May-2019

We appreciate the constructive and insightful comments made by the referees. As proposed, we have substantially changed the manuscript. Please find below our responses to the specific comments made. We would like to note that we made a new figure (Figure 2) as requested, and hence the previously named Figures 3-5 have been renamed to Figures 4-6 (this is indicated in the response every time renamed figures are referenced). We hope that we have fully answered the reviewers' questions and that our replies will help you to make the final decision.

Responses to Editors and Reviewers

21-Jan-2019

Manuscript Number:EJN-2018-11-26016
Action in auctions: neural and computational mechanisms of bidding behavior

Dear Dr Martinez-Saito,

We have now received comments on your article from two experts. They are both overall positive and have mostly requested clarifications and extra illustrations. In addition to the very constructive reviewers' comments, we request that you take the following comments into account.

You need to limit your conclusions to what was measured and you should phrase the presentation of the results to avoid the classic 'absence of evidence is not evidence of absence' error, for instance in this sentence:

EJN European Journal of Neuroscience FENS Federation of European Neuroscience Societies

“Reaction times (RT) did not differ significantly across market types: 11.2±3.6s, 11.1±3.8s, and 11.8±3.8s for SC, NC, and BC, respectively.”

You can see examples of phrasing for frequentist results here:

https://discourse.datamethods.

https://peerj.com/preprints/26

– We limited our conclusions and modified the phrasing of frequentist results as suggested, and wherever applicable, we replaced the term statistical significance with descriptive statements of p-values and effect sizes.

You could simply mention that the (mean?) RT were similar, and report the estimates, confidence intervals and p values, without dichotomising the results. Please also describe the measure of central tendency used for reaction times.

– The sentence was modified as suggested.

It can be found, as it originally was, on page 23: “Mean reaction times (RT) were similar across market types (mean±s.d.): 11.2±3.6s, 11.1±3.8s, and 11.8±3.8s for SC, NC, and BC, respectively.”

If you dichotomise p values based on an arbitrary threshold, then there is no such thing as “weakly significant”. However, it is fine to treat p values as continuous measures of compatibility between the data and the model.

“we found a significant correlation” - we encourage you to remove the terms significant / non-significant as they do not add any information, they only provide the illusion of certainty.

– We eliminated all instances of such wording, as suggested.

All correlations should be illustrated using scatterplots.

Figure 1B: please add plots of the pairwise differences in another panel, so that readers can get assess effect sizes.

– As requested, we added a plot of pairwise differences in Figure 1B (lower panel).

Figures 2A 4B: please use scatterplots instead of bar graphs.

– We have some misgivings about what would scatterplots in these instances represent. The mentioned figures (now 3A and 5B) are unlike for example Figure 3B (previously 2B), because they do not show two variables covarying between subjects, but instead they display the variability (by means of errorbars) of two (Figure 3A, prev. 2A) or several (Figure 5B, prev. 4B) one-dimensional variables (cf. Fig. 5 of Fouragnan et al. (2017)). It is unclear to us what would the requested scatterplots axis would denote. Maybe boxplots or violin plots were meant? Since we would happily comply to improve to the text, we would appreciate a more detailed explanation.

Figures 3, 4A, 5: please provide non-thresholded maps. Here are examples and code:
Allen, E.A., Erhardt, E.B. & Calhoun, V.D. (2012) Data visualization in the neurosciences: overcoming the curse of dimensionality. Neuron, 74, 603-608.
mialab.mrn.org/datavis/
https://github.com/bramzandbel
https://github.com/spinicist/n

– Thank you for the suggestion. We substituted the maps with dual-coded images (in all, now renamed, Figures 4, 5A, 6), and we believe this vastly enriched the illustrative value of the figures.

Also, and this is an important issue, we strongly encourage you to post your data online (using figshare for instance) or to indicate in the article why you cannot share them. Please also provide your analysis code.

– We uploaded s**ource code implementing artificial bidders of DL- and RL-type, model fits, and simulation results to GitHub (https://github.com/mmartinezsaito/action-in-auctions). This is indicated in the Data accessibility section (page 34).**
We also formatted fMRI and behavioral data to comply with the BIDS specification in order to upload them to the online database OpenNeuro (*https://openneuro.org/*). However, they are not available yet because we are having trouble with the uploading process. We will make them available as soon as the problem is solved.

Please also attend to the following issues identified by our editorial office:
1. Separate figure legends will be needed
2. Please ensure that you provide a text and a figure file for the Graphical Abstract (as detailed in the instructions below).
3. You will need to provide more detailed information regarding the ethical committee that approved the work

– We clarified that the protocol was performed in accordance with the Declaration of Helsinki with approval of the University Review Board of Higher School of Economics (page 6).

4. Your bibliography must be carefully checked and put into EJN style at this stage

When revising the manuscript, please embolden or underline major changes to the text so they are easily identifiable and DO NOT leave 'track change' formatting marks in your paper. When carrying out your revisions please refer to the checklist below and visit the EJN author guidelines at www.ejneuroscience.org

When finalized, please upload your complete revised manuscript onto the website, as a Word file (.doc, or .docx). Please also ensure that a complete set of tables and figures is included as separate files, even if these have not changed from the originals. At this stage it is necessary to provide high resolution figures. Please see important instructions below.

Please go into https://mc.manuscriptcentral.c- Author Centre - manuscripts with decisions where you will find a 'create a revision' link under 'actions'. We ask that you please indicate the way in which you have responded to the points raised by the Editors and Reviewers in a letter. Please upload this response letter as a separate Word (.doc or PDF) file using the file designation "Authors' Response to Reviewers" when uploading your manuscript files. Please DO NOT submit your revised manuscript as a new one. Also, please note that only the Author who submitted the original version of the manuscript should submit a revised version.

If you are able to respond fully to the points raised, we would be pleased to receive a revision of your paper within 12 weeks.

Thank you for submitting your work to EJN.

Kind regards,

John Foxe & Paul Bolam
co-Editors in Chief, EJN

Reviews:

Reviewer: 1

EJN European Journal of Neuroscience FENS Federation of European Neuroscience Societies

Comments to the Author
Martinez-Salto et al. present an interesting study examining the neural correlates of biding strategies under different types of context varying in numbers of sellers and buyers. The authors propose to use a new model - the directional learning model - to describe behavior, which ultimately seems to do a better job at explaining participants responses than more classical model-free or model-based RL models. Their results indicate a complementary job of the RL-like signal (RPE) and the DL learning signal in the striatum which is interesting while others areas seem to mediate early strategies. The analyses are comprehensive and the results appear robust and carefully described. However, I think there remains some minor work to do to provide clarity for the reader on the key question that the paper is seeking to answer.

1) The BICs on the model fit are quite convincing (Figure 2a). The best DL model is shown to fit the average profit score in Fig 2c but this is a coarse data feature, and from the data it is not clear what differentiate the best DL from the others and the model-based models. Is it possible to visualize the data feature(s) that distinguishes the model predictions? E.g. presumably the models make different predictions as a function of the elapsed time since the beginning of a market type? Seeing as the critical imaging findings rest on model-derived value regressors, the conclusion that the DL model is the best model of behaviour needs to be shored up by additional analysis beyond a comparison of BIC scores.

– We followed Reviewer 1 suggestion by providing plots of the initial action value functions and preferred bids of RL and DL-type models respectively, and also the evolution across trials of action-value function maxima and preferred bids.

We added on page 25: "To visualize differences in predictive behavior, we performed posterior predictive checks of the the best-fitting algorithms of RL and DL type (Figure 2B), i.e., we simulated replicated data under the fitted models and then compared these to the observed data (Gelman & Hill, 2007). This confirmed that DL-type algorithms were able to learn rapidly profitable bids in each market type (Figure 2B, lower right), whereas RL-type algorithms learned slowly, even when furnished with ad-hoc rules to learn faster (as indicated by the maxima of action-value functions; Figure 2B, lower left)."

2) Similarly, since the DL model is relatively unknown in the field and could be of interest for a larger audience, is it possible to have an intuition in a graph of its core features?

– We made a new figure (Figure 2A) where we provide intuitive illustrations and analogies and abridge the features of DL-type models in juxtaposition with RL-type models.

3) Finally, what is the relationship between the pseudo-RPE and the RPE derived from the other models? Would these correlate?

EJN European Journal of Neuroscience FENS Federation of European Neuroscience Societies

– We added a small clarification about what would RPEs based on the ill-fitting algorithms stand for. In brief, in order for the RPEs to be useful as predictors of behavior, they should be grounded on relaible expected values, which is not the case for RL algorithms in our task.

It can be found on page 27: "We reasoned that it is unsound to search for correlates of variables extracted from the ill-fitting RL algorithms (for example, their RPEs would be grounded on possibly very inaccurate expected values and thus be poor indicators of learning behavior)".

4) What's the relationship between the market discrimination index (MDI) and the DL compliance scores? It looks like they might be related from Fig. 2B&C.

– We included a brief argument as to why DL compliance and MDI should be correlated: the gist is that it is precisely the ability to quickly learn differences among market types (of which MDI is a rought index) what makes DL a profitable strategy.

On page 24: "Thus, in our task, better market discrimination is associated on average with higher profit. Because in our task DL compliance score predicts profit precisely due to its ability to adapt quickly by caching preferred bids between market types, and thence finessing discriminability among market types, it should be as well correlated with MDI. "

5) The figures lack consistency and clarity in presentation. The task on figure 1A is hard to read while B&C others present different font size for similar elements. What is the error bar on the BIC score on figure 2A? In figure 3, the titles have a very low resolution. And what is the Z or T score for the statistical maps?

– We redrew all figures at 300 dpi resolution and chose consistent fonts in Figure 1. The errorbar in Figure 3A (prev. 2A) corresponds to 95% CI. This was indicated before at the bottom of the figure legend, and now we moved it to its corresponding section (A). The thresholding of the SPMs was indicated in the main text, but following Reviewer 1's request we included this information also in the figure legends.

6) Why did the authors decide to remove 5 out of 8 regressors in their model when doing the orthogonalization of OUTCOME_DS and OUTCOME_RPE? Do the results remain unchanged if they use the exact same model (aside the orthogonalization)?

– We wanted to conduct an analysis specific to RPE/DS to focus on learning signals and their orthogonalized counterparts. Therefore, we only included the regressors associated to learning signals during OUTCOME, such that the order of the columns corresponding to pseudo-RPE and DS signals was varied between the two regressor matrices we constructed specifically to study the learning phase, as

described in the text. The results didn't change when adding the other regressors unrelated to learning processes, and we modified the text to reflect this.

We added an explanation on page 20: "(including other regressors irrelevant to learning processes didn't change the results)."

7) Would it be possible to see - as an illustration (scatter plot) - the correlation between the between-subject differences in MDI and the activity in the prefrontal cortex during processing of outcomes (OUTCOME stage, Figure 3B)? I find it unclear from the figure to see if it is a positive or negative correlation. What about the average profit score? Does that also correlate?

– We believe Reviewer 1 here meant to write Figure 3C (now renamed Figure 4C). We plotted and uploaded the requested scatterplots in file 'fig4c_supp.tif'. Although a positive trend between signal at OUTCOME and MDI and average profit is visible, both correlation coefficients were not statistically different from zero at the 0.05 level (t=1.17, df=25, p=0.25; and t=0.82, df =25, p=0.42, respectively for MDI and average profit). However, we note that in Figure 4C (prev. 3C) the displayed activities correspond to inter-subject correlations between MID and the contrast ACCEPTED-REJECTED at OUTCOME stage.

8) I am unsure whether the OUTCOME_DS is only the valence of OUTCOME_pseudo_RPE (+1 positive RPE and -1, negative RPE). It looks like it is from the methods. Is that right? In this case, orthogonalizing the two (OUTCOME_pseudo_RPE with respect to OUTCOME_DS) ends up creating almost an unsigned RPE and keeping the valence DS regressor intact. An unsigned ROE is then pretty much a surprise (how far from expectation) signal. It would be interesting to discuss the results in light of recent work showing multiple valence and surprise representation in the striatum (Fouragnan et al., 2017, figure 5).

– Thank you very much for providing this important reference. Yes, OUTCOME_DS corresponds to the valence of OUTCOME_pseudo-RPE. As suggested, we discussed the commonalities of our study with Fouragnan and colleagues' by emphasizing the existence of concurrent representations of learning signals.

On page 30: "One possibility is that both learning systems operate concurrently, perhaps distributed over a broader network, as recent work that showed multiple distributed RPE valence and surprise representations (Fouragnan et al., 2017). In connection with this, it is interesting to note that the pseudo-RPE signal orthogonalized w.r.t. the DS signal is conceptually analogous to an unsigned RPE (RPE "surprise"), that DS is analogous to RPE valence, and that both signals were found to pertain to a common network for the computation of learning signals, in agreement with Fouragnan et al. (2017)."

EJN European Journal of Neuroscience FENS Federation of European Neuroscience Societies

Reviewer: 2

Comments to the Author
Martinez-Saito and co-authors report on an ambitious computational cognitive neuroscience study into the neural correlates of sequential bidding behaviour. The manuscript is very well written, the methodology used adheres to the highest standards in the field, and the evaluation of the behavioural and neuroimaging data is extensive. In brief, the authors find that directional learning (DL) models outperform conventional Rescorla-Wagner-type reinforcement learning models in their ability to describe human choice behaviour in sequential bidding. Moreover, the authors identified correlates of DL model trial-by-trial parameters in the striatum, evidence for the encoding of the action space in the posterior parietal cortex, and a correlation of bid adjustment with neural activity in the DLPFC. I have a number of suggestions to further improve the clarity of this manuscript, which the authors may consider for a minor revision of their work.

Task description
I think that the choice environment could be characterized in a more formal and more quantitatively explicit manner. Specifically, participants performed the double auction task in three different conditions (seller competition, no competition, buyer competition) against the responses of pre-recorded human subjects replayed by a computer. I would think that the adaptive bidding behaviour of the participants can be appreciated more comprehensively, if the readers had information about these pre-recorded choices. For example, were the pre-recorded bids quantitatively similar to the data observed for the current participant cohort? Ideally, and because the authors already refer to concepts from the theory of Markov decision processes on occasion, the authors would describe their task by a tuple (S,A,R,p(s_t|s_{t-1}, p(r_t|s_t))) of state, action, and reward spaces, as well as state transition and state-conditional reward probabilities, respectively, with which the participants either interacted objectively (i.e., from the perspective of the authors) or subjectively (i.e., based on the cognitive modelling assumptions of the authors). This would allow readers to easier understand the task and appreciate the (in)appropriateness of certain choice policies used in the behavioural models.

– We followed both suggestions: we added our post-experimental observation (done but not mentioned previously) that prerecorded buyers and the current buyers cohort behaved indistinguishably, and we supplemented the task description with and addendum using the language of Markov decision models.

The new text can be found on pages 7-8: “The task can be formalized within the framework of Markov decision processes as a 4-tuple $(S, A, R, T = p(s_i \mid s_j), R = p(r \mid s, a))$, denoting respectively the set of states consisting of the three market types *S={“SC”, “NC”, “BC”};* the set of actions consisting of all possible bids $A=\{0, 0.1, \ldots, 10\}$; the state transitions probabilities, which are trivial because each market type evolves independently ($p(s_i \mid s_j) = \delta_{ij}$, where s_i denotes market type i and δ_{ij} is the Kronecker delta); and the state-action-conditional reward probabilities $R = p(r \mid s, a)$, which depend on the behavior of the

EJN European Journal of Neuroscience FENS Federation of European Neuroscience Societies

opponents such that *r = 10 - a* (where *a* is the action or bid) if the bid a overbids all opponent bids and ask prices depending on the market type, and *r = 0* otherwise.
Crucially, here the behavior of the opponent is unknown a priori and can be assume to be internally represented as a probability distribution over competitor buyers' bids and sellers' ask prices. The form of this probability distribution is a decisive factor determining bidding behavior, but under the modest assumption that subjects believe there exists a natural clearing price characteristic of each market with a reasonably small variance, we can model it approximately as a Gaussian distribution with center at the estimated clearing price. For example, in a simple auction with one buyer and one seller, the buyer would hold an estimate of the (possibly varying) seller's ask price, and would try to maximize profit by choosing the lowest possible bid that doesn't fall below the seller's ask price. Then, a strategy consisting of simply tracking competitor buyers' bids and sellers' ask prices would motivate a DL-type and not RL-type algorithm (see Figure 2A)."

And in page 10: "In a post-experiment check, we found that the behavior of buyers in the prerecorded data was indistinguishable from the behavior of buyers in the current participant cohort."

Behavioural modelling
Model-free RL: I think the value Q_t(b|m) for the state-action-value function Q_t : S \times A -> \mathbb{R} would better be denoted by Q_t(b,m), as the vertical line is easily confused with probabilistic conditioning, which is not meant here. The same holds for the action probability P(b|m), where the potential confusion is even more severe, as a probability is denoted.

– We followed the suggestion, but by renaming Q_t(b|m) as Q_m,t instead of Q_t(b,m) in order to avoid confusion with the usual notation for joint probability distributions.

The authors report that they initialized the state-action-value function with a Beta distribution based on the subject-pooled first trial bids. This is somewhat confusing. A Beta distribution is defined on the interval [0,1]. The action space, on the other hand, appears to have consisted of the discrete set A = {0,0.1,0.2,…,10} with cardinality 101. Do the authors mean that they scaled the action space into the continuous interval [0,1] or that they used a multinoulli distribution over the discrete action space? It would also be helpful to visualize the initial and final state-action-value functions to appreciate the degree of adaptation throughout the task.

– Thank you for this remark. Our description was indeed unclear, and thus we added a clarifying paragraph to define precisely in which way we used the Beta distribution, i.e. by scaling the action space into the continuous interval [0,1] (we rescaled both its support and range). Additionally, we included in Figure 2B a plot of the initial action-value functions (the final action-value functions were almost identical due to the inability of the RL algorithm to learn quickly).

EJN European Journal of Neuroscience FENS Federation of European Neuroscience Societies

On page 12: “Conventional Beta distributions are parameterized with two shape parameters, are defined on the real interval [0, 1], and their definite integral equals 1, but in our task the action-value space spanned the interval [0, 10] and the sum of action-values is not constrained. Thus, we rescaled both the support (from [0, 1] to [0, 10]) and the range of the Beta distribution to yield a usable prior for the Q_m functions.”

Model-based RL with counterfactual learning: the action conditional update rule on p. 12 is slightly confusing. First, I am not clear on the domain of the state-action value-function used here, with respect to the state-action-value function used for the model-free RL. In the current setting, the function has three arguments, i

– (The text we received was cut short at this point, but we believe we understood the argument) We cleared up the description of the algorithm, corrected a notation typo, and redesignated the Q function for market *m* as Q_m instead of Q(|m). The domain of both action-value functions is exactly the same. We hope the new notation is clearer.

The changes can be found on page 13:

“If b accepted: for all $i < b$, $Q_{m,t+1}(i) \leftarrow Q_{m,t}(i)$

for all $i \geq b$, $Q_{m,t+1}(i) \leftarrow Q_{m,t}(i) + \alpha \left(r - Q_{m,t}(i)\right)$

If b rejected: for all $i \leq b$, $Q_{m,t+1}(i) \leftarrow Q_{m,t}(i) + \alpha \left(0 - Q_{m,t}(i)\right)$

for all $i > b$, $Q_{m,t+1}(i) \leftarrow Q_{m,t}(i)$,”

DL as value-free, model-based learning: The authors note that DL is suitable if there exists a unique optimal action. It may be helpful to mention the form of this unique optimal action for the current choice environment or at least provide a reference for its formal proof of existence. Naively, I would assume the optimal actions in the different markets scenarios are different, but exist, and depend on the expected bids of competitors, expected seller’s price, as well as the specified loss and disagreement outcomes.

– We rewrote this section to comply with your recommendation. We expounded the idea of optimal bid and how it depends on other players, and emphasized that it should be close to the unique minimal bid below which all other bids are rejected.

The text can be found on pages 13-14: “DL requires an a priori knowledge about the structure of the environment, and it is suitable only under specific circumstances: the space of feasible actions should be a totally ordered set (all its elements satisfy some mutual relationship by which they can be unambiguously characterized by a single index), and there should exist a unique optimal action associated to each game

environment at each time point. Our task satisfies these conditions: bids are ordered respect to a magnitude of interest (bid size or bid value), and in each market scenario, there is a noisy clearing price whose average may or may not exhibit a time-dependent drift. If the game environment is not stationary, DL will track the optimal price with some lag. The optimal action will depend in general on the utility functions of players (which comprises social preferences), and on the choice randomness of competitor buyers and sellers, but assuming that typical sellers (buyers) entertain reasonably stationary ask prices (bids), the optimal bid should be approximately the unique minimal bid below which all other bids are rejected. ”

I think a figure that conveys the central assumptions of each behavioural model and provides some exemplary learning trajectories could increase appreciation of the different model types.

– We made a new figure (Figure 2) where we provide (Figure 2A) intuitive illustrations and analogies and abridge the features of DL-type models in juxtaposition with RL-type models. We also show posterior predictive simulations of DL and RL algorithms (Figure 2B). We also discussed Figure 2B in the text.

We added on page 25: “To visualize differences in predictive behavior, we performed posterior predictive checks of the the best-fitting algorithms of RL and DL type (Figure 2B), i.e., we simulated replicated data under the fitted models and then compared these to the observed data (Gelman & Hill, 2007). This confirmed that DL-type models were able to learn rapidly profitable bids in each market type (Figure 2B, lower right), whereas RL-type models, even when furnished with ad-hoc rules to learn faster, learned slowly (as indicated by the maxima of action-value functions; Figure 2B, lower left).”

I strongly encourage the authors to upload their behavioural modelling code as well as the aggregated anonymized behavioural data to a data sharing platform such as the OSF or Github. As a standard for documenting the data, the BIDS format for behavioural data may be suitable.

– We uploaded s**ource code implementing artificial bidders of DL- and RL-type, model fits, and simulation results to GitHub (https://github.com/mmartinezsaito/action-in-auctions). This is indicated in the Data accessibility section (page 34).**

Functional neuroimaging data

I appreciate that the authors attempted to estimate the power of their study, but I think the approach used (Cheng and Schwarzman, 2015) does not really relate to the statistical tests reported later, as these would have to be performed with the same statistical test implied by Cheng and Schwarzman (for a detailed review of the SPM p-values relied on in the current study, see e.g. https://arxiv.org/abs/1808.040). I would suggest omitting the power calculation.

– We followed the suggestion, and omitted the calculation.

European Journal of Neuroscience FENS Federation of European Neuroscience Societies

In the GLM analysis, the authors introduce the preferred bid value (PBV). I think it would help to introduce this central variable for the model-based analysis of the fMRI data already in the discussion of the DL model to make the link between the behavioural and functional imaging models more explicit.

–Thank you for the suggestion. We totally agree with the suggestion, and moved this subsection to the DL algorithm description.

The moved text straddles pages 14-15 now: "In the DL scheme, the variables tracking currently estimated action values are not conventional expected values, but rather an estimation of the value of the maximum reward obtainable, namely the preferred bid value (PBV). Computing an expectation over a probability distribution of values associated with actions is not possible in a DL algorithm because there is no action value function over which a measure can be integrated, but PBVs can be interpreted as a rough equivalent of the conventional expected values of RL algorithms. Thus, it is possible to defined a *pseudo-RPE signal* as a RPE where the expected value is assumed to be the currently preferred bid."

I would suggest to supplement the GLM-based results figures with a depiction of an annotated exemplary design matrix, as this is the easiest way to communicate the designs used.

– As suggested, we included an exemplary design matrix in Figure 5 (prev. 4) to illustrate the correspondence between the order of first and second parametric modulators and non-orthogonalized and orthogonalized regressors, respectively.

The sentence "Temporal serial correlations in fMRI data were removed with the (SPM12) autoregressive AR(1) model to satisfy the parameter estimation routine (ReML) assumptions" is askew. SPM12's ReML routine uses a covariance component estimation approach to estimate the degree of serial correlations. The estimated covariance matrix is then used to "whiten" the data, i.e. to remove serial correlations. The aim of this is to have the whitened data conform to the sphericity assumption necessary for the use of standard z, T, and F distribution in parametric inference (see e.g. https://doi.org/10.3389/fnins.).

– Thank you very much for pointing this out. We have corrected the sentence by paraphrasing the correct description, that you kindly provided, of the routine.

On page 20: "Temporal serial correlations in fMRI data were removed using the residuals covariance matrix estimated by the restricted maximum likelihood routine in SPM12 to satisfy the sphericity assumption needed for doing inference (Starke & Ostwald, 2017)."

Unfortunately, the description of the analysis involving fitting fMRI data to the learning algorithm (p. 19-20, Results p. 24) is opaque to me. Which fMRI data features does this refer to? The trialby-trial nature of the description of the analysis seems to imply a trial-wise feature, but the extraction of this feature is not explicitly mentioned. This part of the manuscript should be improved significantly.

– We appreciate thas this was brought to our attention, since the description was indeed unclear and inconsistent. The variables we were referring to were the preferred bid value, and the predictions errors (DS and pseudo-RPE).

We fully rewrote the paragraph, which can be seen now on page 22: “To localize potential brain regions involved in the computation of the economic transactions, we assessed on a trial-by-trial basis the correlations between neural data and model proxy variables. The dataset comprising all the game sequences from all subjects was used to fit the parameters of each learning algorithm. The fitting process was informed by plausible assumptions about the players strategies, such as initializing prior bid values (see section “Computational algorithms of adaptive learning” for details). We selected the best algorithm based on BIC scores. Then, we derived time series of expected values (PBV) and prediction error (DS, pseudo-RPE) signals from each of the learning algorithms by making each of the artificial bidding agents to enact human subjects behavior. This entailed pitting the artificial bidders against the same sequences of stimuli that the human subjects played against, and in each trial computing the proxy variables (PBV, pseudo-RPE, DS) furnished by their underlying learning algorithm, conditioned on the fact that they selected the same bids as the human subject they were enacting. ”

Table 2 and its caption are somewhat confusing. First, why do the authors report only z-scores, not T-values? The uncorrected peak voxel p-value reported in the penultimate column is commonly evaluated based on the respective test statistic, which would be a T-value in the current case (the cluster extent p-value is applied to T-values under the assumption of high degrees of freedom, in this sense, reporting the Gaussianized z-scores makes sense, although I think that most researchers, including myself, commonly assume that their study degrees of freedom are high enough, which in the current study with n = 27 is also the case).

– We had no specific reason other than to use a more interpretable statistic. However, we agree that following the convention to report t-statistics is more sensible, and changed the Tables accordingly.

Second, the authors write that here and below all voxels are significant at least at p <0.001, uncorrected for the whole brain. Does this refer to the cluster-defining threshold? From the methods part my understanding was that the cluster-defining threshold was set to p < 0.05 FWE corrected.

– Thank you for indicating this issue. $p < 0.001$ uncorrected for the whole brain refers here to the criterion threshold for reporting neural activity. The methods section was indeed unclear due to an oversight. We reported activity at $p < 0.001$ uncorrected for activity in the ROIs, including the orthogonalized contrasts in striatum, but normal contrasts in striatal areas were reported at FWER-corrected $p < 0.05$ level. We modified the methods sections to reflect this unambiguously.

The new text is on page 21: "Activations of learning signals (DS and pseudo-RPE) in the striatum and outside regions of interest (ROI) were reported at a voxel-level threshold of $p<0.05$ after voxel-based family-wise error rate (FWER) correction. Activations were reported in other ROIs, and also in orthogonalized contrasts (i.e., the second parametric modulator regressor for a given event in the design matrix) when they exceeded a voxel-level primary threshold of whole-brain $p<0.001$ uncorrected and a cluster-level extent threshold of 10 voxels. Because such scheme yields a FWE-corrected p-value of 0.6~0.9 (Eklund et al., 2016), it was used only in regions that previous studies consistently reported to be involved in value-based decision making and mentalizing in interactive play games (Barraclough et al., 2004; Bartra et al., 2013; Rilling et al., 2004; Carter et al., 2012), in internal representation of the number line and manipulation of arithmetic objects (Dehaene et al., 2003, 2004). These ROIs were orbitofrontal cortex, frontopolar and dorsolateral prefrontal cortex, anterior cingulate cortex, medial prefrontal cortex, and temporo-parietal junction. Cluster-defining thresholds for all types of activity inference were appropriately set at $p=0.001$ (Eklund et al., 2016; Flandin & Friston, 2017). Brain regions are displayed on a standard MNI template. All clusters from all figures are listed in Tables 2, 3, and 4. Thresholded cluster edges are indicated with black contour lines. Activation maps were dual-coded (Allen et al., 2012), where significance level and effect size were represented by means of color saturation and hue, respectively, with MATLAB code from Zandbelt (2017)."

Again, with respect to the penultimate column of the table: is this the corrected or uncorrected peak-voxel p-value? If it is the uncorrected p-value, it would be redundant, because the entire map is thresholded at $p < 0.001$ according to the caption.

– This column is indeed redundant because it's the uncorrected p-value, so we duly removed it.

Finally, with respect to the statement "cluster-level inference alone may yield inflated false positives (Eklund, 2016, Flandin and Friston 2017)" - I think the bottom line of both studies cited is that for an appropriately high cluster-forming threshold (e.g. corresponding to an uncorrected p-value < 0.001), the FWE corrected p-value for cluster extent is approximately correct for the resting state fMRI data analysed in these studies.

– We removed this sentence from the Table and instead added and explanation about the used SPM thresholds in the methods section, as mentioned above in the response to the question "Second, the authors write ...".

Finally, I would also encourage the authors to share the functional neuroimaging data in a standardized format, either openly, if possible based on ethical constraints, or in a protected format using data use agreements.

– **We formatted fMRI and behavioral data to comply with the BIDS specification in order to upload them to the online database OpenNeuro (*https://openneuro.org/*). However, they are not available yet because we are having trouble with the uploading process. We will make them available as soon as the problem is solved.**

We hope that we have satisfactorily responded to all comments and we are looking forward to your decision.

Yours sincerely,

Mario Martinez-Saito, *Institute of Cognitive Neuroscience*

Rodion Konovalov, *Research Center of Neurology*

Michael A. Piradov, *Research Center of Neurology*

Anna Shestakova, *Institute of Cognitive Neuroscience*

Boris Gutkin, *Ecole Normale Superieure*

Vasily Klucharev, *Institute of Cognitive Neuroscience*
